# Binding of tryptophan and tryptophan-containing peptides in water by a glucose naphtho crown ether

**DOI:** 10.3762/bjoc.21.42

**Published:** 2025-03-10

**Authors:** Gianpaolo Gallo, Bartosz Lewandowski

**Affiliations:** 1 Laboratory of Organic Chemistry, ETH Zürich, Vladimir-Prelog-Weg 3, 8093 Zürich, Switzerland

**Keywords:** amino acids, macrocyclic receptors, molecular recognition, monosaccharides, tryptophan

## Abstract

Tryptophan fulfills a plethora of important functions in nature both in its free form and as a component of peptides and proteins. Selective binding of tryptophan is therefore important for diagnostic and medicinal applications. Recently, we reported a glucose naphtho crown ether which is a chemoselective receptor for the esters of aromatic amino acids, in particular tryptophan, in water. Herein, we demonstrate that the same compound also binds free tryptophan selectively in aqueous media. Furthermore, it is capable of binding to tryptophan within model tripeptides. The naphthalene functionality in the glucose-derived receptor enables the study of guest binding using fluorescence spectroscopy.

## Introduction

**Figure 1:**
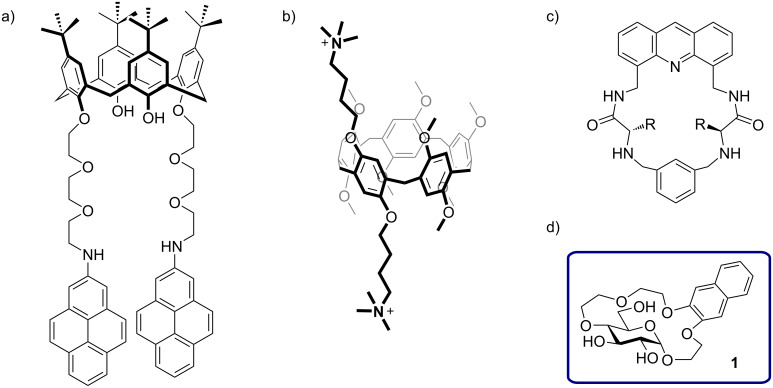
a–c) Examples of synthetic receptors for selective binding of tryptophan in aqueous media, taken from refs. [14–16]; d) glucose-based naphtho crown ether **1** – a chemoselective receptor for aromatic amino acid esters in water.

Tryptophan plays a crucial role in a variety of biological processes [1–2]. For example, it is critical for electron transfer in proteins [3] and is a key component of several membrane proteins as well as short antimicrobial peptides [4–5]. Tryptophan also serves as a substrate to produce hormones such as serotonin or melatonin [6–7]. Due to the biological relevance of tryptophan synthetic receptors for this amino acid are highly sought after [8]. From a biological perspective it is especially desirable to achieve selective binding of tryptophan in water [9]. However, the development of selective amino acid receptors in aqueous environments is challenging since it requires a combination of hydrophobic and polar interactions for binding [10–12]. As a result, the number of reported selective receptors for tryptophan in aqueous media is limited (Figure 1a–c) [13–18]. In particular, receptors which bind tryptophan residues in peptides are rare [19–20]. Recently, we developed a glucose-based naphtho crown ether **1** (Figure 1d) which binds amino acid methyl esters with aromatic side chains chemoselectively in water [21]. Crown ether **1** is particularly suited for the sensing of Trp methyl ester, which it binds with a higher affinity than the Phe and Tyr esters. Additionally, the binding of Trp-OMe results in a highly efficient fluorescence quenching of the naphthalene unit in the receptor. Herein, we report that receptor **1** can be used for the binding and fluorescent sensing of tryptophan as well as tryptophan residues in short peptides in water.

## Results and Discussion

Having previously demonstrated that **1** binds tryptophan methyl ester in water [21] we wanted to investigate whether it is also suitable for the binding of free, unprotected tryptophan. To determine, if **1** binds tryptophan selectively over other amino acids we also studied phenylalanine, glutamic acid, lysine, and leucine as guests. We chose these amino acids to verify, how the character of their side chains (Phe – aromatic, Glu – anionic, Lys – cationic, Leu – hydrophobic) affects their binding by the receptor. Additionally, to probe whether the chiral glucose backbone of the receptor allows to achieve selective binding of one enantiomer of tryptophan over the other we used both ʟ- and ᴅ-tryptophan as guests. The binding of amino acids in H_2_O by **1** was investigated by ITC measurements and titration experiments monitored by fluorescence spectroscopy. The results are summarized in Table 1.

**Table 1:** Binding affinities of receptor **1** to amino acids determined by ITC experiments and fluorescence measurements in H_2_O.

Entry	Guest	*K*_a_ (ITC)^a^	*K*_a_ (Fl)^b^

1	H-ʟ-Trp-OH	990 ± 310^c^	1340 ± 30
2	H-ʟ-Leu-OH	350 ± 160	330 ± 10
3	H-ʟ-Phe-OH	400 ± 130	620 ± 20
4	H-ʟ-Glu-OH	360 ± 140	460 ± 10
5	H-ʟ-Lys-OH	570 ± 220	1060 ± 20
6	H-ᴅ-Trp-OH	660 ± 270	1030 ± 20

^a^*K*_a_ values are reported in M^−1^; ^b^Fl = fluorescence measurements; ^c^fitting errors are reported for both types of measurements.

Receptor **1** binds tryptophan with the highest affinity among the investigated amino acids (entry 1, Table 1). The *K*_a_ of the complex between **1** and H-ʟ-Trp-OH is also considerably higher than the values observed for the complexes of previously reported crown ether-type receptors with zwitterionic amino acids in water [22]. The affinity of **1** towards leucine, bearing a hydrophobic side chain, is over 4 times lower than towards tryptophan (entry 2, Table 1). Pleasingly, phenylalanine, which alike Trp contains an aromatic side chain is also bound by **1** with a more than two-fold lower affinity (entry 3, Table 1). Glu with an anionic side chain, is bound by **1** almost three-fold weaker than Trp. The binding affinity of **1** towards Lys with a cationic side chain is the second highest among the tested amino acids and approximately 30% lower than towards Trp (entry 5, Table 1). Thus, the glucose-derived naphtho crown ether **1** displays selectivity towards the binding of tryptophan over other amino acids in water. Additionally, **1** displays a slight preference towards ʟ-Trp over its ᴅ-enantiomer (entries 1 and 6, Table 1).

Since tryptophan fulfills many of its biological functions as a component of peptides and proteins [2–5], we also probed, if **1** can recognize tryptophan residues within peptide sequences. For this purpose, we prepared six model tripeptides **2**–**7** consisting of one tryptophan and two alanine residues. We varied the position of the tryptophan in the peptide sequence, N-terminus in **2**, middle of the chain in **3**, and C-terminus in **4** to check if this affects their binding by **1**. To probe the importance of the N-terminal amino group in the peptides for binding we prepared the derivatives of peptides **2**–**4** bearing an acetyl-capped N-terminus (compounds **5**–**7**). As a control we also studied tripeptide **8** containing exclusively alanine residues. The binding of tripeptides was again studied by ITC and fluorescence spectroscopy and the results are summarized in Table 2.

**Table 2:** Binding affinities of receptor **1** to peptides **2**–**8** determined by ITC experiments and fluorescence measurements in H_2_O.

Entry	Guest	*K*_a_ (ITC)^a^	*K*_a_ (Fl)^b^

1	H-TrpAlaAla-NH_2_ (**2**)	2440 ± 950^c^	2820 ± 90
2	H-AlaTrpAla-NH_2_ (**3**)	2890 ± 780	2630 ± 70
3	H-AlaAlaTrp-NH_2_ (**4**)	1830 ± 400	2060 ± 40
4	Ac-TrpAlaAla-NH_2_ (**5**)	1650 ± 650	1380 ± 40
5	Ac-AlaTrpAla-NH_2_ (**6**)	1840 ± 410	1910 ± 60
6	Ac-AlaAlaTrp-NH_2_ (**7**)	1720 ± 500	1980 ± 60
7	H-AlaAlaAla-NH_2_ (**8**)	650 ± 220	640 ± 10

^a^*K*_a_ values are reported in M^−1^; ^b^Fl = fluorescence measurements; ^c^fitting errors are reported for both types of measurements.

**Figure 2:**
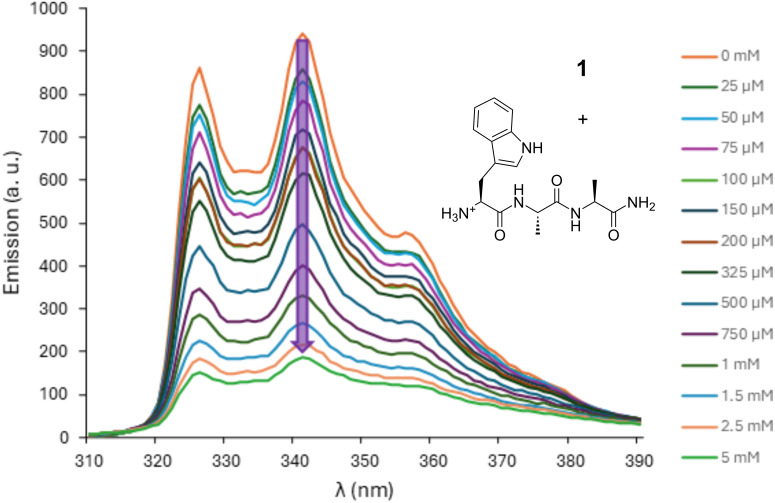
Fluorescence emission spectra of receptor **1** (25 μM in H_2_O) upon addition of increasing amounts of tripeptide **2**. The concentration of the guest corresponding to each curve is indicated on the right. The arrow marks the quenching of naphthalene fluorescence during the titration.

Receptor **1** binds tripeptides **2**–**4** with considerably higher affinities than tryptophan itself. In the case of peptides **2** and **3** where the Trp residue is at the N-terminus or in the middle of the chain the *K*_a_ is approximately 2× higher than for the amino acid binding (entries 1 and 2, Table 2). The binding affinity of **1** towards peptide **4** is around 1.5 times higher than for the free amino acid (entry 3, Table 2). The acetylated analogues of peptides **2** and **3** – compounds **5** and **6**, are bound by **1** notably weaker, with 1.8× and 1.3× lower affinities respectively (entries 4 and 5, Table 2). Peptide **7**, the acetylated analogue of **4**, is bound by **1** with essentially the same affinity as the non-acetylated variant (entry 6, Table 2). Peptide **8**, consisting of three alanine residues, is bound by the glucose naphtho crown ether with a significantly lower affinity, 3–4 times weaker than the Trp-containing peptides **2**–**4** (entry 7, Table 2). For each of the peptides **2**–**7** containing a Trp residue in the sequence binding to the receptor resulted in significant quenching of its fluorescence (Figure 2 and Figures S1–S5 in Supporting Information File 1).

**Figure 3:**
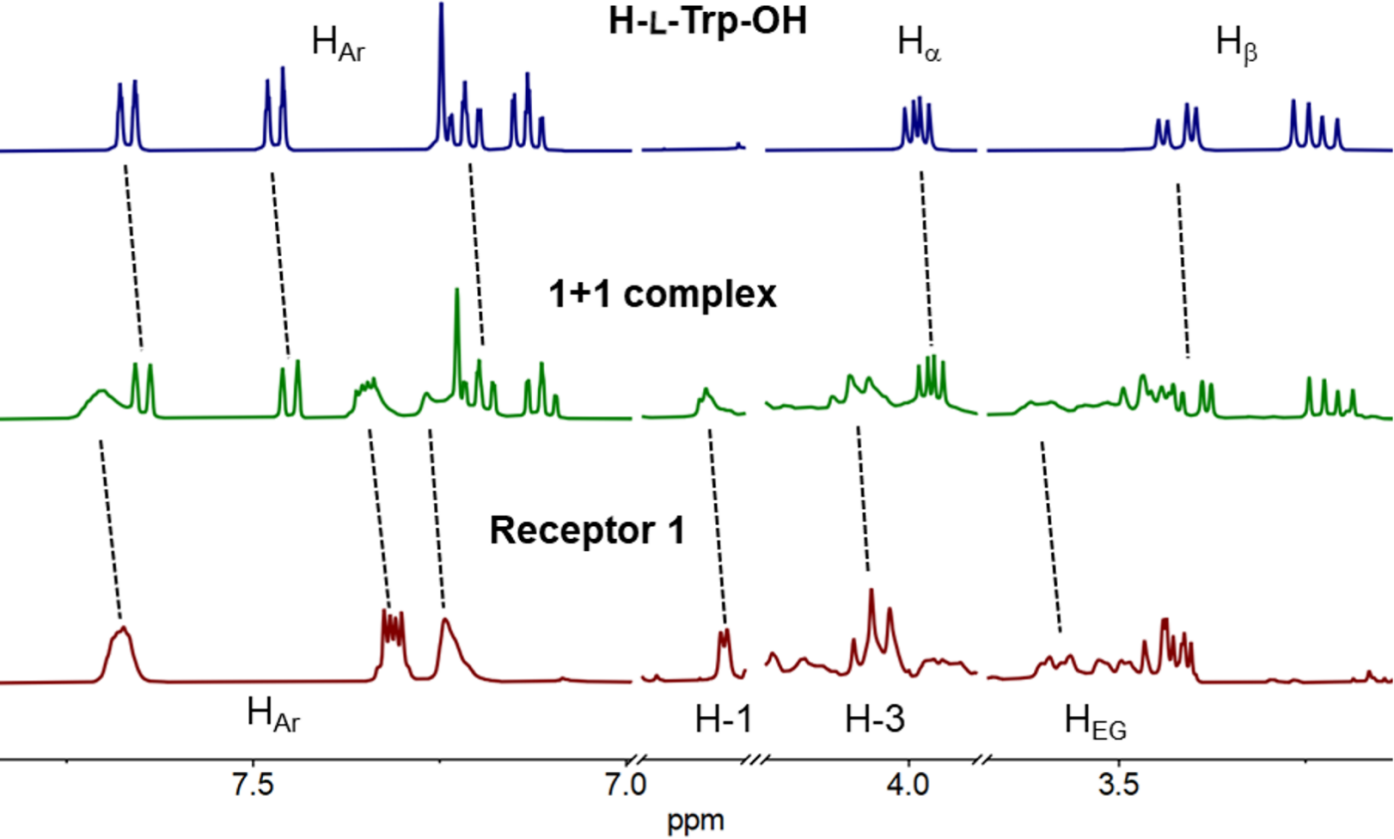
^1^H NMR (D_2_O) stacked plot: top – H-Trp-OH; middle – H-Trp-OH + receptor **1** (1:1); bottom – receptor **1**; the dotted black lines indicate shifts of the proton signals upon complex formation.

To gain a deeper insight into the binding mode of tryptophan and Trp-containing peptides by **1** we performed NMR measurements on the 1:1 host–guest complexes in D_2_O. Upon binding of tryptophan by **1** a significant upfield shift of the aromatic protons and a slightly smaller shift, also upfield, of the α and β-Trp protons was observed (Figure 3). The aromatic protons of **1** shifted downfield and a marginal downfield shift of several crown ether protons as well as H-1 and H-3 of the glucopyranose was also observed. These observations suggest that the main interaction between **1** and H-Trp-OH is the π–π stacking between the naphthalene unit of **1** and the indole moiety in the amino acid. This hypothesis is also supported by the significant quenching of the fluorescence of the naphthalene unit in **1** upon addition of increasing amounts of H-Trp-OH to the solution. Additionally, cation–π interactions between the ammonium cation of the guest and the naphthalene of the host as well as Coulombic interactions between the crown ether and the ammonium could be involved. Furthermore, the ITC measurements revealed that the binding of **1** with both enantiomers of H-Trp-OH is predominantly entropy-driven suggesting that the hydrophobic effect plays an important role in the binding process.

**Figure 4:**
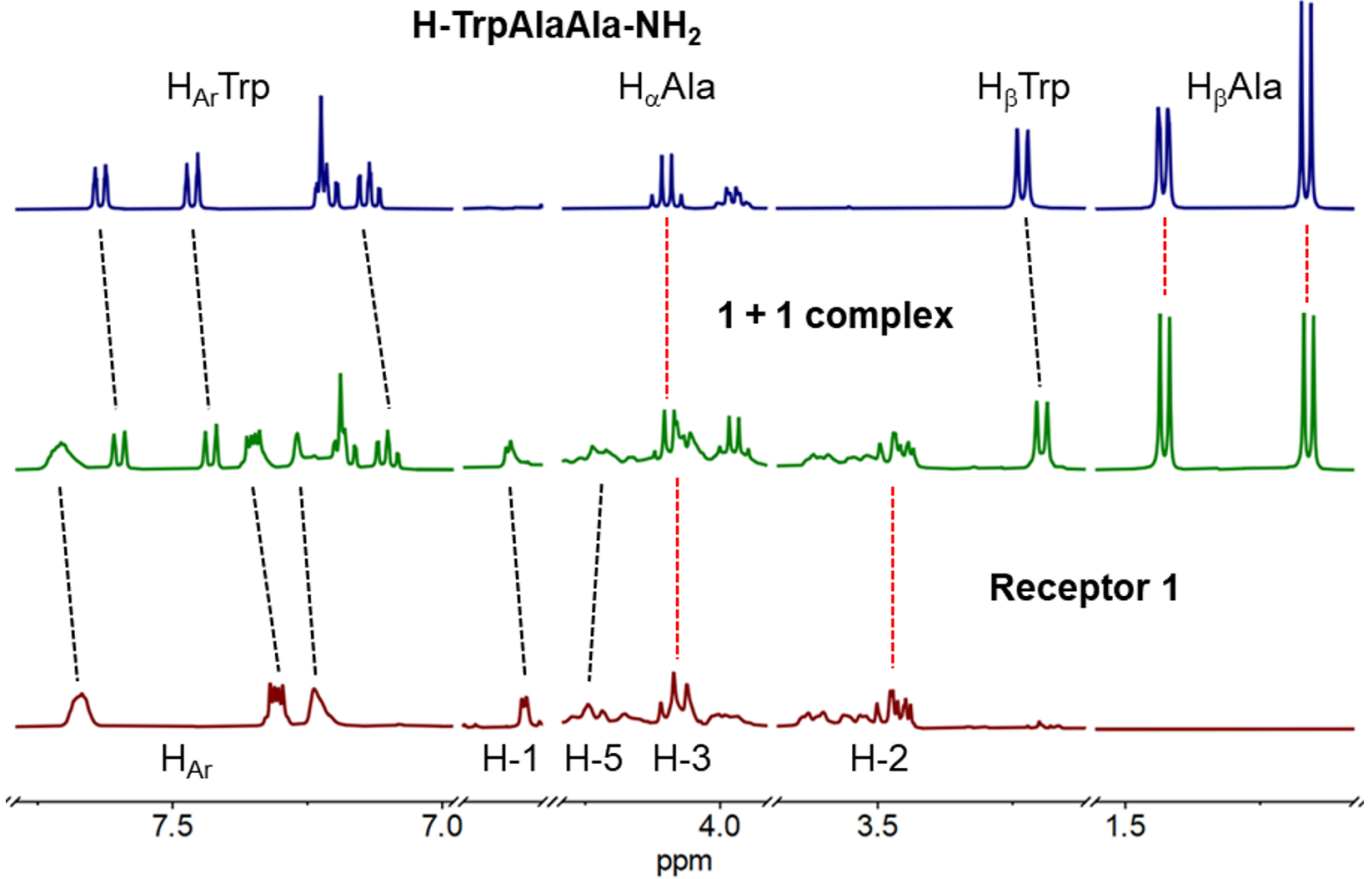
^1^H NMR (D_2_O) stacked plot: top – H-TrpAlaAla-NH_2_ (**2**); middle – **2** + receptor **1** (1:1); bottom – receptor **1**; the dotted black lines indicate shifts of the proton signals upon complex formation and dotted red lines indicate lack of signal shift.

In the case of tripeptides **2** and **3** upon binding by **1** strong upfield shifts of the aromatic protons and a small upfield shift of the β-protons of tryptophan were observed (Figure 4 and Figure S6 in Supporting Information File 1). Interestingly, basically no shifts of the alanine protons occurred. As for the receptor, downfield shifts of the naphthalene protons were observed. For the H-TrpAlaAla-NH_2_ (**2**)@**1** complex an additional downfield shift of H-1 and upfield shift of H-5 was detected. In the H-AlaTrpAla-NH_2_ (**3**)@**1** complex, H-2 and H-6 shifted downfield (Figure S6 in Supporting Information File 1).

**Figure 5:**
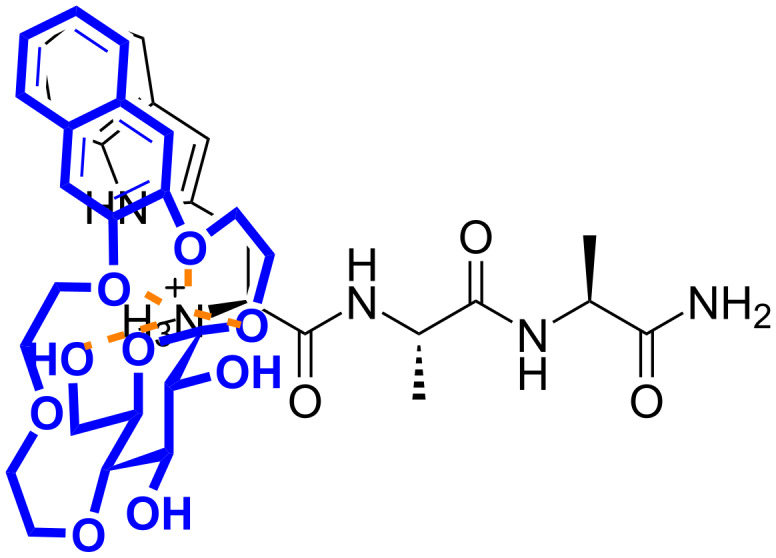
Proposed binding mode of receptor **1** to tripeptide **2**.

These observations suggest that receptor **1** binds selectively to the Trp residues in the studied tripeptides and the binding occurs predominantly through π–π interactions. This conclusion is supported by the considerably lower affinity of **1** to peptide **8**, lacking a Trp residue, and by the fluorescence measurements where a strong quenching of the naphthalene fluorescence upon addition of each of the Trp-containing tripeptides **2**–**7** was observed. For peptides **2** and **3** interactions of the receptor with the N-terminal ammonium cation are likely also involved in the binding process (Figure 5). This is as well indicated by the lower binding affinity of **1** to the acetylated analogues of peptides **2** and **3**. In the case of peptide **4**, acetylation of its N-terminus did not affect the stability of the complex with receptor **1**. This suggests that **1** interacts with the N-terminal ammonium in **4** to a much lesser extent. The receptor could instead potentially form H-bonds via its OH functionalities with the amide groups adjacent to the Trp residue in the peptide. The favourable negative entropy of the interaction between **1** and the tripeptides **2**–**7** suggests a significant contribution of the hydrophobic effect to binding in each case.

## Conclusion

We have shown that glucose-based naphtho crown ether binds tryptophan selectively over other amino acids in water. The same receptor is also capable of binding to tryptophan residues within peptide chains which was demonstrated on six model tripeptides. Owing to the presence of the naphthyl moiety in the receptor the binding process can be monitored by fluorescence spectroscopy. These results pave the way for further investigations on the binding and fluorescent sensing of tryptophan within biologically relevant peptides by our glucose-based receptor. Furthermore, they open exciting opportunities for the development of other monosaccharide-based selective receptors of amino acids in aqueous environments.

## Supporting Information

File 1 Materials and instruments, synthetic protocols for preparation of peptides **2**–**8**, details of ITC, and fluorescence and NMR experiments.

## Data Availability

All data that supports the findings of this study is available in the published article and/or the supporting information of this article.
