# Supplementary material for: Binding of tryptophan and tryptophan-containing peptides in water by a glucose naphtho crown ether
Source: Beilstein J Org Chem. 2025 Mar 10;21:541–6. doi: 10.3762/bjoc.21.42 (PMC11897655; doi:10.3762/bjoc.21.42)

BEILSTEIN JOURNAL OF ORGANIC CHEMISTRY

Supporting Information

for

Binding of tryptophan and tryptophan-containing peptides in water by a glucose naphtho crown ether

Gianpaolo Gallo and Bartosz Lewandowski

Beilstein J. Org. Chem. **2025,** *21,* 541–546. doi:10.3762/bjoc.21.42

Materials and instruments, synthetic protocols for preparation of peptides 2–8, details of ITC, and fluorescence and NMR experiments

License and Terms: This is a supporting information file under the terms of the Creative Commons Attribution License (https://creativecommons.org/licenses/by/4.0). Please note that the reuse, redistribution and reproduction in particular requires that the author(s) and source are credited and that individual graphics may be subject to special legal provisions.
The license is subject to the *Beilstein Journal of Organic Chemistry* terms and conditions: (https://www.beilstein-journals.org/bjoc/terms)

Table of contents

1. Supporting figures for the manuscript S2
2. Materials, methods and instruments S5
3. Synthesis of receptor **1** and tripeptides **2**–**8** S6
4. Titration experiments S9
   4.1 ITC – raw data, fitted curves and result tables S9
   4.2 Fluorescence - spectra, fitted curves and result tables S15
5. NMR spectra – receptor **1** and tripeptides **2**–**8** S29

1. Supporting figures for the manuscript

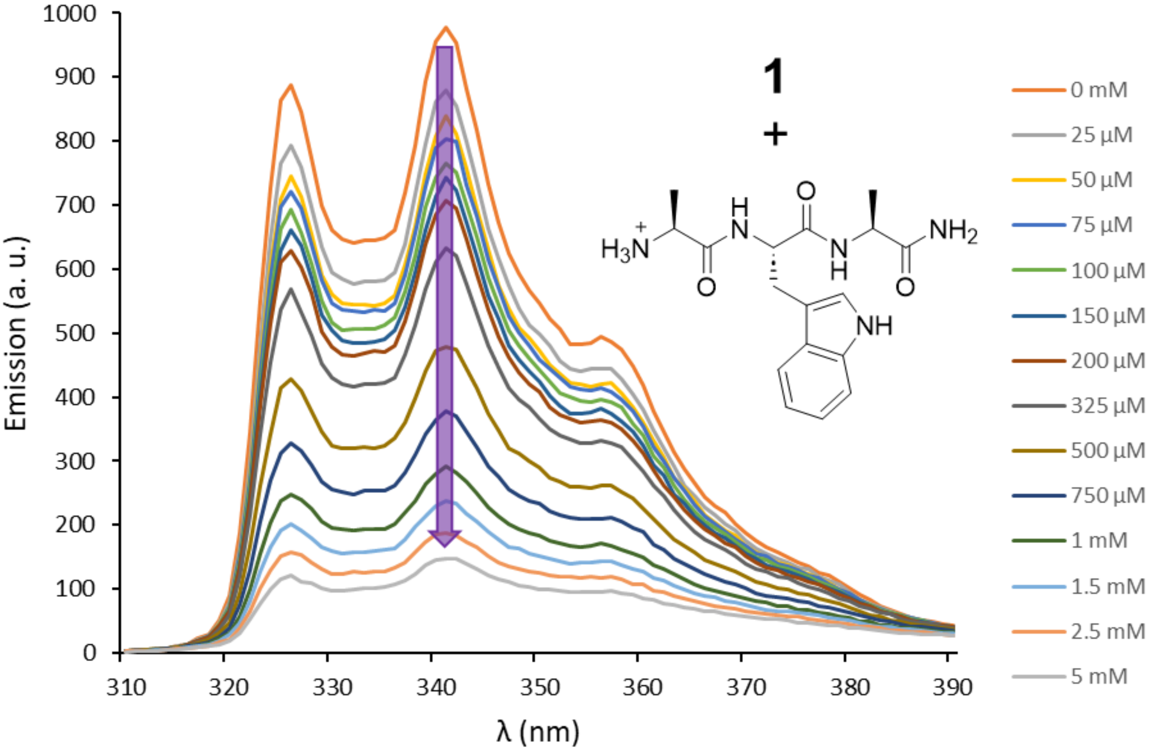

Figure S1 Fluorescence emission spectra of receptor **1** (25 µM in H_2O) upon addition of increasing amounts of tripeptide **3**. The concentration of the guest corresponding to each curve is indicated on the right. The arrow marks the quenching of naphthalene fluorescence during the titration.

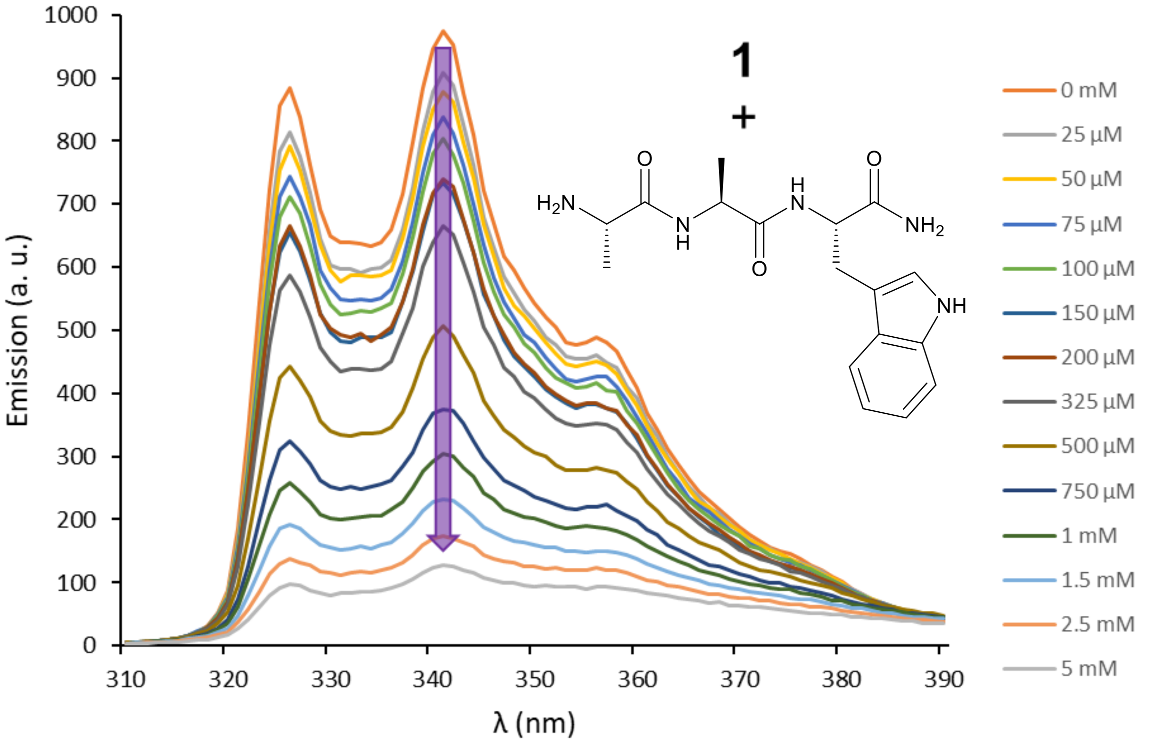

Figure S2 Fluorescence emission spectra of receptor **1** (25 µM in H_2O) upon addition of increasing amounts of tripeptide **4**. The concentration of the guest corresponding to each curve is indicated on the right. The arrow marks the quenching of naphthalene fluorescence during the titration.

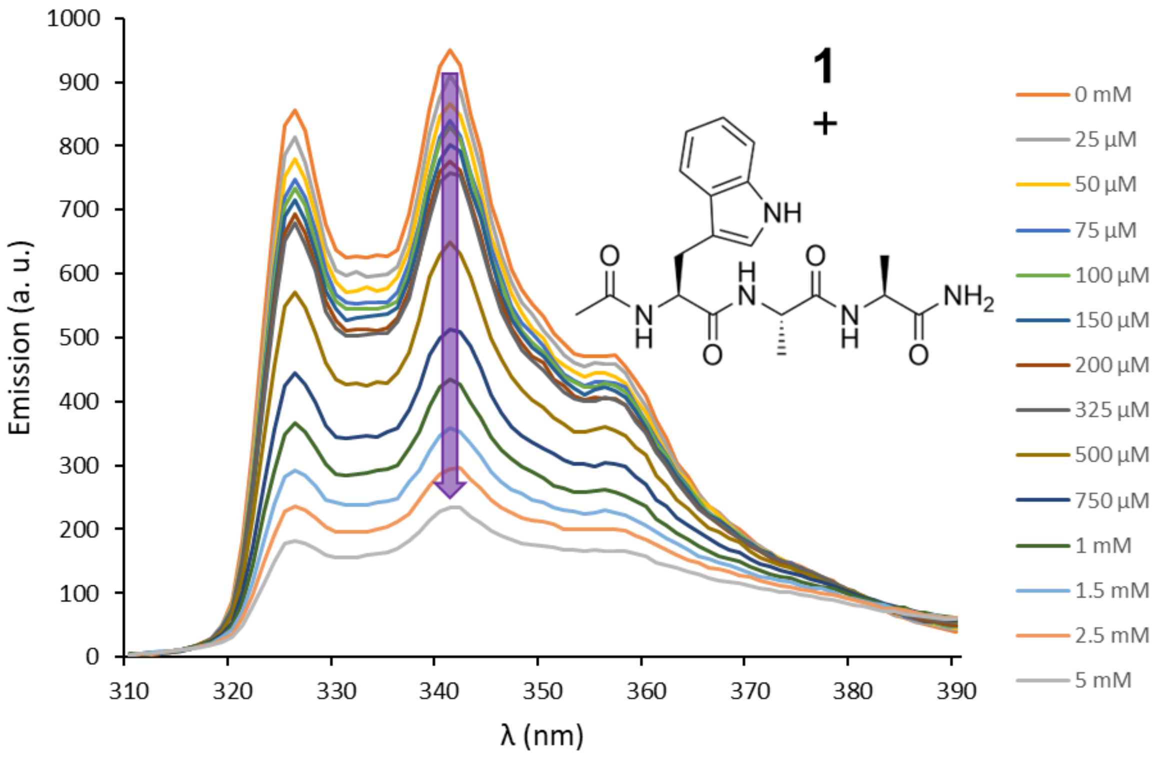

Figure S3 Fluorescence emission spectra of receptor **1** (25 µM in H_2O) upon addition of increasing amounts of tripeptide **5**. The concentration of the guest corresponding to each curve is indicated on the right. The arrow marks the quenching of naphthalene fluorescence during the titration.

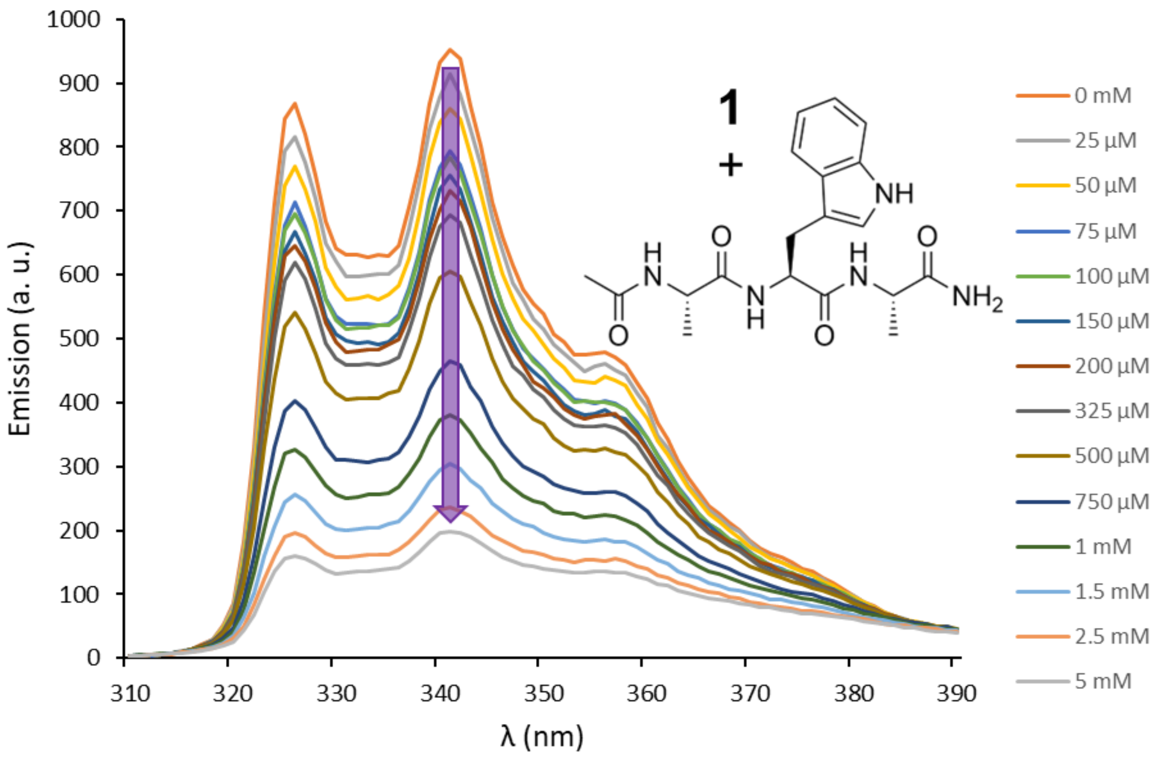

Figure S4 Fluorescence emission spectra of receptor **1** (25 µM in H_2O) upon addition of increasing amounts of tripeptide **6**. The concentration of the guest corresponding to each curve is indicated on the right. The arrow marks the quenching of naphthalene fluorescence during the titration.

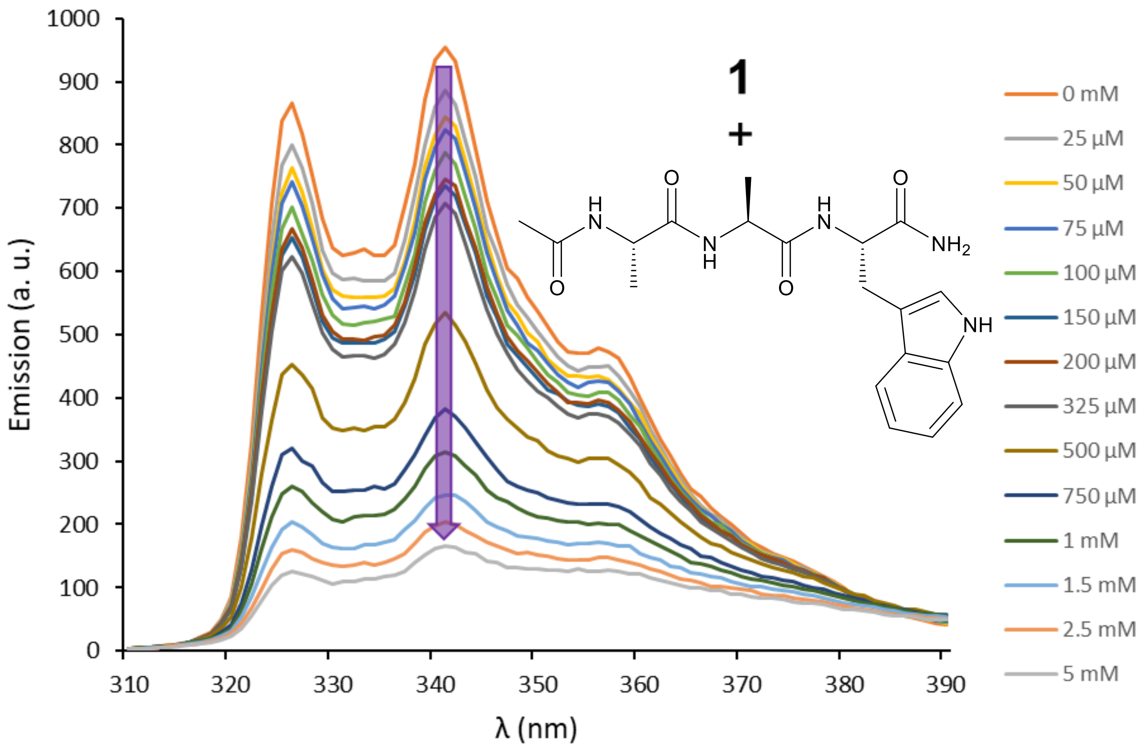

Figure S5 Fluorescence emission spectra of receptor **1** (25 µM in H_2O) upon addition of increasing amounts of tripeptide **7**. The concentration of the guest corresponding to each curve is indicated on the right. The arrow marks the quenching of naphthalene fluorescence during the titration.

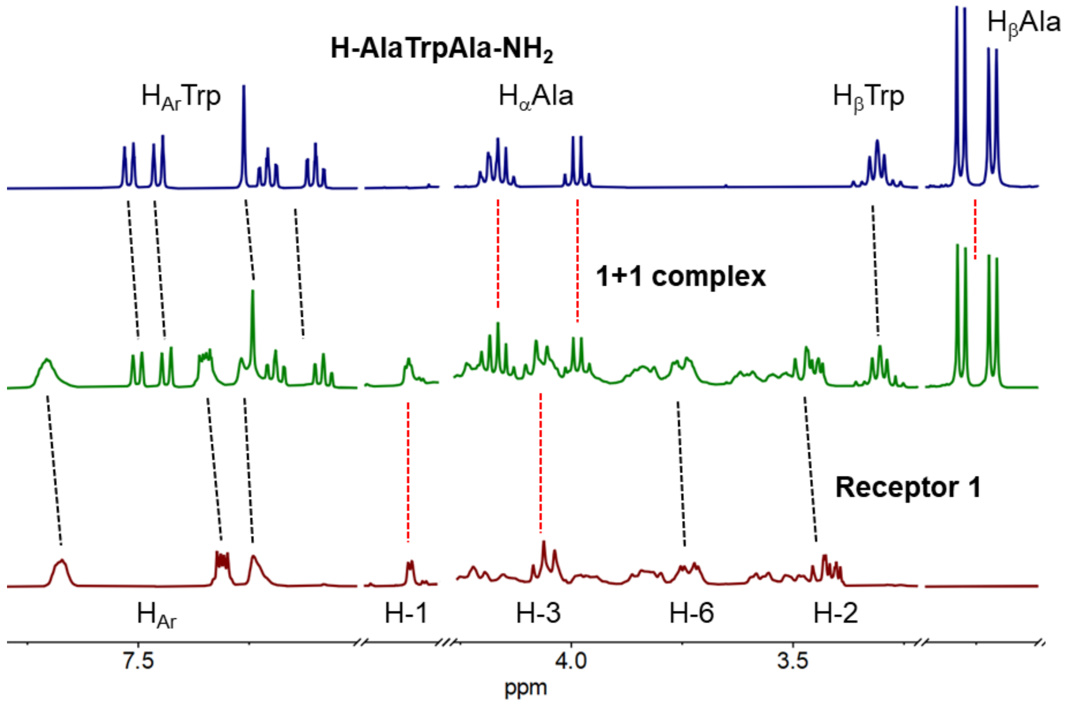

Figure S6 ^{1}H NMR (D_2O) stacked plot: top – H-AlaTrpAla-NH_2 (**3**); middle – H-AlaTrpAla-NH_2 + receptor **1** (1:1); bottom – receptor **1**; the dotted black lines indicate shifts of the proton signals upon complex formation, dotted red lines indicate lack of signal shift.

2. Materials, methods and instruments

Materials

Solvents and reagents were of the highest commercially available grade and used without further purification. They were purchased from Sigma Aldrich, Fischer Scientific, Fluka, Bachem, BioMatrix, Biotage, IRIS Biotech, Protein Technologies and Acros Organics. Solvents used for MPLC were HPLC-grade quality.

Solid phase peptide synthesis (SPPS)

SPPS was performed using the Fmoc-strategy and Rink amide-PS as resin. Amino acids and coupling reagents were purchased from Bachem, IRIS Biotech and Protein Technologies. For automated peptide synthesis, a SYRO Robot peptide synthesizer (Biotage, Sweden) controlled by the SyroXP Peptide software version 2.0.126 (MultiSynTech GmbH, Germany) was used.

Thin-layer chromatography (TLC)

TLC was conducted on aluminium sheets coated with silica gel 60 F_{254} (Merck) using UV fluorescence (254 and 366 nm). Analytical grade solvents were used.

Liquid chromatography–mass spectrometry (LC–MS)

Analytical reversed phase HPLC (RP-HPLC) was performed on a Dionex UHPLC, Ultimate 3000. Reprosil gold 120 C_{18} (150 × 4 mm, 5 µm) with a flow of 0.5 mL/min was used as the analytical column. Two different solvents were used. Solvent A was assigned to be pure acetonitrile and solvent B was a mixture of 1% acetonitrile and 0.1% TFA in Milli-Q pure water. The mass analysis was performed on an amaZone speed ion trap mass analyzer (Bruker, USA).

Nuclear magnetic resonance (NMR) spectroscopy

1D and 2D NMR spectra were recorded on 400, 500, and 600 MHz Ultrashield spectrometers (Bruker, USA). ^{1}H NMR chemical shifts (δ_H) are quoted in parts per million (ppm) and coupling constants (J) are quoted in hertz (Hz). Abbreviations for NMR data are s (singlet), d (doublet), t (triplet), q (quartet), m (multiplet).

High-resolution mass spectrometry (HRMS)

High-resolution electrospray ionization (HRMS–ESI) spectra were measured on a Bruker maXis spectrometer.

Lyophilization

An Alpha 2-4 LD plus lyophilizator (Christ, Germany) was used.

Isothermal titration calorimetry (ITC)

ITC experiments were carried out in a MicroCal PEAQ-ITC (Malvern), at 25 °C, 750 rpm, high feedback and 10 µcal/s as reference power, using Milli-Q water as a solvent.

Fluorescence spectroscopy

Fluorescence emission spectra were recorded on a Cary Eclipse Fluorescence Spectrophotometer (Varian Inc.) in Milli-Q water, at 25 °C.

3. Synthesis of receptor **1** and tripeptides **2**–**8**.

Receptor **1** was prepared following the previously reported procedure [Chak, M. H.; Schnurr, M.; Lewandowski, B. *Eur. J. Org. Chem.* **2023,** *26,* e202300305. doi:10.1002/ejoc.202300305].

Tripeptides **2**–**8** were prepared using automated SPPS as outlined in the materials and methods section. Upon completion of the synthesis the peptides were cleaved off solid support. For this purpose, the resin containing the attached tripeptide was agitated for 1 h in a mixture of TFA/CH_2Cl_2/TIPS (90:7.5:2.5) and the solution was collected by filtration. The filtrate was concentrated to a small volume under reduced pressure and the product was precipitated from cold Et_2O. The white solid was isolated by centrifugation of the suspension and decanting the supernatant. The solid was triturated with Et_2O twice and the residual white solid was dried under a stream of nitrogen, dissolved in H_2O and lyophilized to obtain the desired peptides (each isolated as a white amorphous solid).

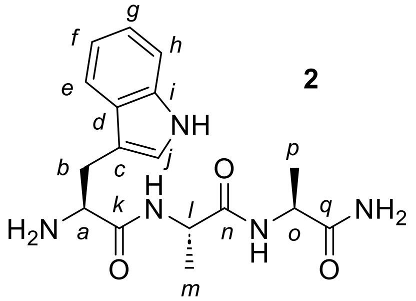

2

^{1}H NMR (400 MHz, MeOD) δ 7.70 (dt, *J* = 7.8, 1.1 Hz, 1H, H_e), 7.40 (dt, *J* = 8.1, 1.0 Hz, 1H, H_h), 7.25 (s, 1H, H_j), 7.16 (ddd, *J* = 8.2, 7.0, 1.2 Hz, 1H, H_g), 7.08 (ddd, *J* = 8.0, 7.0, 1.1 Hz, 1H, H_f), 4.42 (q, *J* = 7.1 Hz, 1H, H_l), 4.34 (q, *J* = 7.1 Hz, 1H, H_o), 4.19 (dd, *J* = 8.7, 5.6 Hz, 1H, H_a), 3.49 (ddd, *J* = 15.0, 5.5, 0.9 Hz, 1H, H_b), 3.22 (dd, *J* = 15.0, 8.8 Hz, 1H, $H_{b'}$), 1.42 (d, J = 7.1 Hz, 3H, H_m), 1.38 (d, J = 7.2 Hz, 3H, H_p).

^{13}C NMR (101 MHz, MeOD) δ 176.0 (C_q), 172.5 (C_n), 168.6 (C_k), 137.0 (C_d), 126.9 (C_i), 124.3 (C_j), 121.5 (C_g), 118.9 (C_f), 117.7 (C_e), 111.2 (C_h), 106.5 (C_c), 53.4 (C_a), 49.2 (C_l), 48.7 (C_o), 27.4 (C_b), 17.0 (C_p), 16.7 (C_m).

HR-ESI-MS: m/z: 346.1867 $[M+H]^+$, calculated for $C_{17}H_{24}N_5O_3^+$: 346.1874.

3

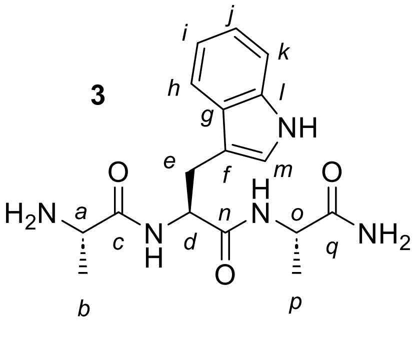

^{1}H NMR (400 MHz, MeOD) δ 7.66 (dt, *J* = 7.7, 1.0 Hz, 1H, H_h), 7.35 (dt, *J* = 8.1, 0.9 Hz, 1H, H_k), 7.17 (s, 1H, H_m), 7.12 (ddd, *J* = 8.2, 7.0, 1.2 Hz, 1H, H_j), 7.04 (ddd, *J* = 8.0, 7.0, 1.1 Hz, 1H, H_i), 4.75 (dd, *J* = 8.2, 6.3 Hz, 1H, H_d), 4.32 (q, *J* = 7.1 Hz, 1H, H_o), 3.89 (q, *J* = 7.0 Hz, 1H, H_a), 3.36 (dd, *J* = 6.4, 0.8 Hz, 1H, H_e), 3.17 (ddd, *J* = 14.6, 8.3, 0.7 Hz, 1H, $H_{e'}$), 1.50 (d, *J* = 7.0 Hz, 3H, H_b), 1.32 (d, *J* = 7.1 Hz, 3H, H_p).

^{13}C NMR (101 MHz, MeOD) δ 175.7 (C_q), 171.9 (C_n), 169.6 (C_c), 136.7 (C_g), 127.2 (C_l), 123.3 (C_m), 121.2 (C_j), 118.5 (C_i), 118.0 (C_h), 111.0 (C_k), 109.3 (C_f), 54.4 (C_d), 48.7 (C_a), 48.6 (C_o), 27.5 (C_e), 17.0 (C_p), 16.2 (C_b).

HR-ESI-MS: m/z: 346.1875 $[M+H]^+$, calculated for $C_{17}H_{24}N_5O_3^+$: 346.1874.

4

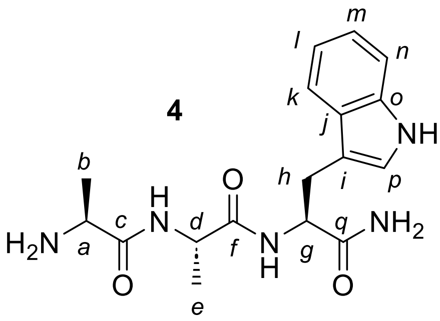

^{1}H NMR (400 MHz, MeOD) δ 7.63 (dt, *J* = 7.8, 1.0 Hz, 1H, H_k), 7.34 (dt, *J* = 8.1, 0.9 Hz, 1H, H_n), 7.14 (s, 1H, H_p), 7.10 (ddd, *J* = 8.1, 7.0, 1.2 Hz, 1H, H_m), 7.03 (ddd, *J* = 8.0, 7.0, 1.1 Hz, 1H, H_l), 4.66 (t, *J* = 6.5 Hz, 1H, H_g), 4.35 (q, *J* = 7.2 Hz, 1H, H_d), 3.89 (q, *J* = 7.1 Hz, 1H, H_a), 3.31 – 3.18 (m, 2H, H_h), 1.37 (d, *J* = 7.1 Hz, 3H, H_b), 1.35 (d, *J* = 7.2 Hz, 3H, H_e).

^{13}C NMR (101 MHz, MeOD) δ 176.9 (C_q), 175.1 (C_f), 173.2 (C_c), 136.6 (C_j), 127.5 (C_o), 123.3 (C_p), 121.1 (C_m), 118.5 (C_l), 118.0 (C_k), 110.9 (C_n), 109.2 (C_i), 53.6 (C_g), 49.8 (C_d), 49.3 (C_a), 27.2 (C_h), 19.6 (C_e), 16.2 (C_b).

HR-ESI-MS: m/z: 346.1869 $[M+H]^+$, calculated for $C_{17}H_{24}N_5O_3^+$: 346.1874.

5

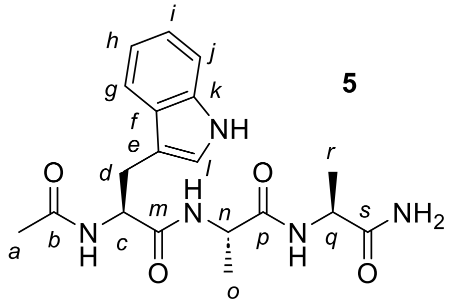

^{1}H NMR (400 MHz, MeOD) δ 7.60 (dt, *J* = 7.9, 1.0 Hz, 1H, H_g), 7.34 (dt, *J* = 8.1, 0.9 Hz, 1H, H_j), 7.17 (s, 1H, H_l), 7.11 (ddd, *J* = 8.2, 7.0, 1.2 Hz, 1H, H_i), 7.03 (ddd, *J* = 8.0, 7.0, 1.1 Hz, 1H, H_h), 4.63 (dd, *J* = 7.7, 5.8 Hz, 1H, H_c), 4.28 (2 x q, *J* = 7.2, 4.5 Hz, 2H, $H_n + H_q$), 3.29 – 3.10 (m, 2H, H_d), 1.95 (s, 3H, H_a), 1.34 (d, *J* = 7.2 Hz, 3H, H_o), 1.26 (d, J = 7.2 Hz, 3H, H_r).

^{13}C NMR (101 MHz, MeOD) δ 176.2 (C_s), 173.1 (C_m), 173.1 (C_p), 172.3 (C_b), 136.6 (C_f), 127.5 (C_k), 123.2 (C_l), 121.1 (C_i), 118.4 (C_h), 117.9 (C_g), 110.9 (C_j), 109.4 (C_c), 54.7 (C_c), 49.4 (C_n), 48.8 (C_q), 27.2 (C_d), 21.1 (C_a), 16.7 (C_r), 16.0 (C_o).

HR-ESI-MS: m/z: 410.1798 $[M+Na]^+$, calculated for $C_{19}H_{25}N_5NaO_4^+$: 410.1799.

6

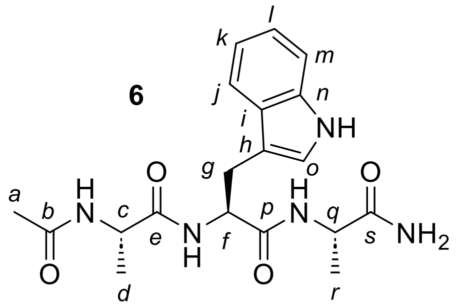

^{1}H NMR (400 MHz, MeOD) δ 7.59 (ddd, *J* = 7.9, 1.2, 0.8 Hz, 1H, H_j), 7.36 (dt, *J* = 8.1, 1.0 Hz, 1H, H_m), 7.16 (s, 1H, H_o), 7.12 (ddd, *J* = 8.2, 7.0, 1.2 Hz, 1H, H_l), 7.05 (ddd, *J* = 8.0, 7.0, 1.1 Hz, 1H, H_k), 4.58 (dd, *J* = 6.7, 5.9 Hz, 1H, H_f), 4.27 (q, *J* = 7.2 Hz, 1H, H_q), 4.19 (q, *J* = 7.2 Hz, 1H, H_c), 3.30 – 3.26 (m, 1H, H_g), 1.83 (s, 3H, H_a), 1.29 (d, *J* = 7.2 Hz, 3H, H_d), 1.19 (d, *J* = 7.3 Hz, 3H, H_r).

^{13}C NMR (101 MHz, MeOD) δ 176.2 (C_s), 174.1 (C_p), 172.4 (C_e), 172.3 (C_b), 136.7 (C_i), 127.4 (C_n), 123.4 (C_o), 121.2 (C_l), 118.7 (C_k), 117.9 (C_j), 111.0 (C_m), 108.9 (C_h), 54.3 (C_f), 49.8 (C_c), 48.9 (C_q), 26.7 (C_g), 20.8 (C_a), 16.3 (C_r), 15.8 (C_d).

HR-ESI-MS: m/z: 410.1804 $[M+Na]^+$, calculated for $C_{19}H_{25}N_5NaO_4^+$: 410.1799.

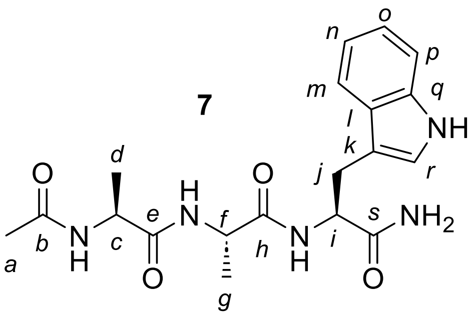

^{1}H NMR (400 MHz, MeOD) δ 7.63 (dt, *J* = 7.9, 1.1 Hz, 1H, H_m), 7.34 (dt, *J* = 8.1, 1.0 Hz, 1H, H_p), 7.13 (s, 1H, H_r), 7.10 (ddd, *J* = 8.1, 7.1, 1.4 Hz, 1H, H_o), 7.03 (ddd, *J* = 8.0, 7.0, 1.1 Hz, 1H, H_n), 4.65 (dd, *J* = 7.8, 5.6 Hz, 1H, H_i), 4.21 (2 x q, *J* = 7.2, 4.3 Hz, 2H, H_c + H_f), 3.31 – 3.20 (m, 2H, H_j), 1.98 (s, 3H, H_a), 1.26 (d, *J* = 7.2 Hz, 3H, H_d), 1.21 (d, *J* = 7.2 Hz, 3H, H_g).

^{13}C NMR (101 MHz, MeOD) δ 175.2 (C_s), 174.3 (C_h), 173.3 (C_e), 172.1 (C_b), 136.6 (C_l), 127.5 (C_q), 123.1 (C_r), 121.1 (C_o), 118.5 (C_n), 118.0 (C_m), 110.9 (C_p), 109.5 (C_k), 53.7 (C_i), 49.7 (C_f), 49.5 (C_c), 27.1 (C_j), 21.0 (C_a), 16.2 (C_g), 15.9 (C_d).

HR-ESI-MS: m/z: 410.1795 $[M+Na]^+$, calculated for $C_{19}H_{25}N_5NaO_4^+$: 410.1799.

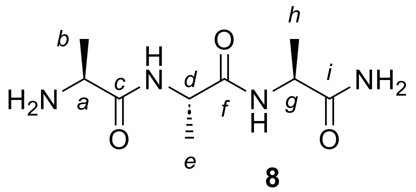

8

^{1}H NMR (400 MHz, MeOD) δ 4.41 (q, *J* = 7.2 Hz, 1H, H_d), 4.35 (q, *J* = 7.1 Hz, 1H, H_g), 3.96 (q, *J* = 7.0 Hz, 1H, H_a), 1.54 (d, *J* = 7.0 Hz, 3H, H_b), 1.42 (d, *J* = 7.2 Hz, 3H, H_e), 1.39 (d, *J* = 7.1 Hz, 3H, H_h).

^{13}C NMR (101 MHz, MeOD) δ 175.9 (C_i), 172.7 (C_f), 169.4 (C_c), 49.1 (C_d), 48.7 (C_a), 48.6 (C_g), 17.1 (C_h), 16.5 (C_e), 16.2 (C_b).

HR-ESI-MS: m/z: 253.1271 $[M+Na]^+$, calculated for $C_9H_{18}N_4NaO_3^+$: 253.1271.

4. Titration experiments.

4.1 Isothermal titration calorimetry (ITC) measurements.

ITC experiments were carried out in a MicroCal PEAQ-ITC (Malvern), at 25 °C, 750 rpm, high feedback and 10 µcal/s as reference power, using MilliQ water as a solvent.

The concentrations of the host and the guest solutions were 1 mM and 30 Mm, respectively. The guest solution was added in 1 µL portions at 2 min intervals.

The data was analysed by fitting to a one set of sites model using the MicroCal PEAQ-ITC Control Software. Dilution control experiments were performed for each guest and the data was subtracted from the data obtained in the titration experiment.

Summary of ITC results (for raw calorimetry data and fitted curves see following pages):

Entry	Guest	K_d (mM)	ΔG (kcal/mol)	ΔH (kcal/mol)	N
**1**	L-Trp	1.01 ± 0.32	-4.08	-0.39	0.7
**2**	L-Leu	2.83 ± 1.27	-3.47	-0.14	1.1
**3**	L-Phe	2.48 ± 0.82	-3.54	-0.27	1.2
**4**	L-Glu	2.78 ± 1.08	-3.48	-0.07	1.2
**5**	L-Lys	1.75 ± 0.68	-3.75	-0.15	1.2
**6**	D-Trp	1.52 ± 0.63	-3.84	-0.21	1.3
**7**	**2**	0.41 ± 0.16	-4.61	-0.71	0.9
**8**	**3**	0.35 ± 0.10	-4.71	-0.44	1.1
**9**	**4**	0.55 ± 0.12	-4.44	-0.34	1.2
**10**	**5**	0.61 ± 0.24	-4.38	-0.32	1.3
**11**	**6**	0.54 ± 0.12	-4.45	-0.38	1.2
**12**	**7**	0.58 ± 0.17	-4.41	-0.19	1.3
**13**	**8**	1.54 ± 0.52	-3.82	-0.12	1.2

H-ʟ-Trp-OH to receptor 1:

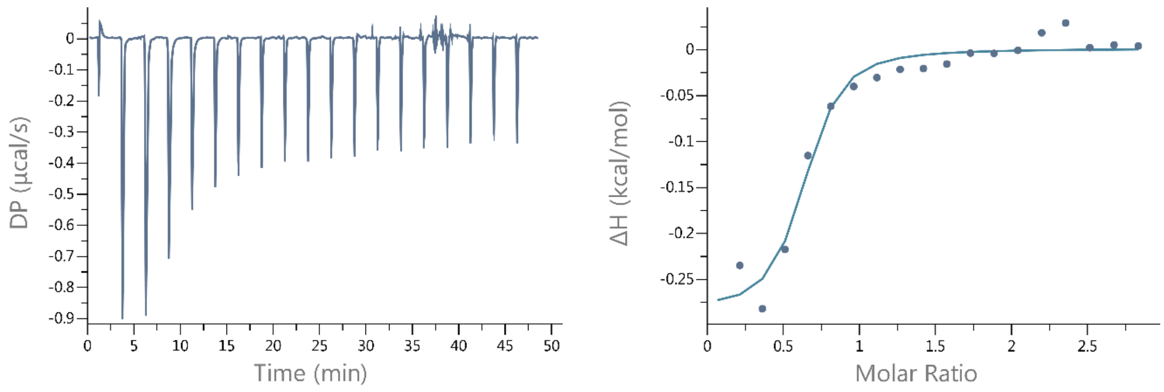

H-ʟ-Leu-OH to receptor 1:

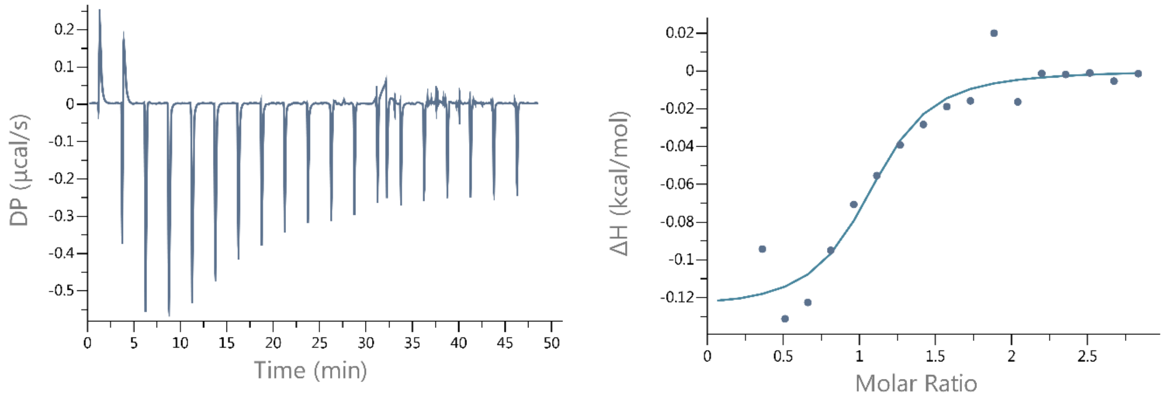

H-ʟ-Phe-OH to receptor 1:

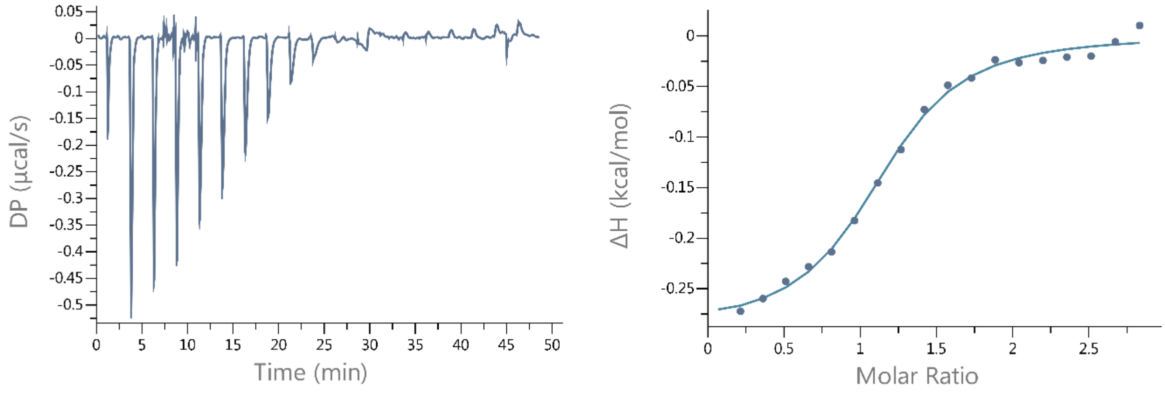

H-ʟ-Glu-OH to receptor 1:

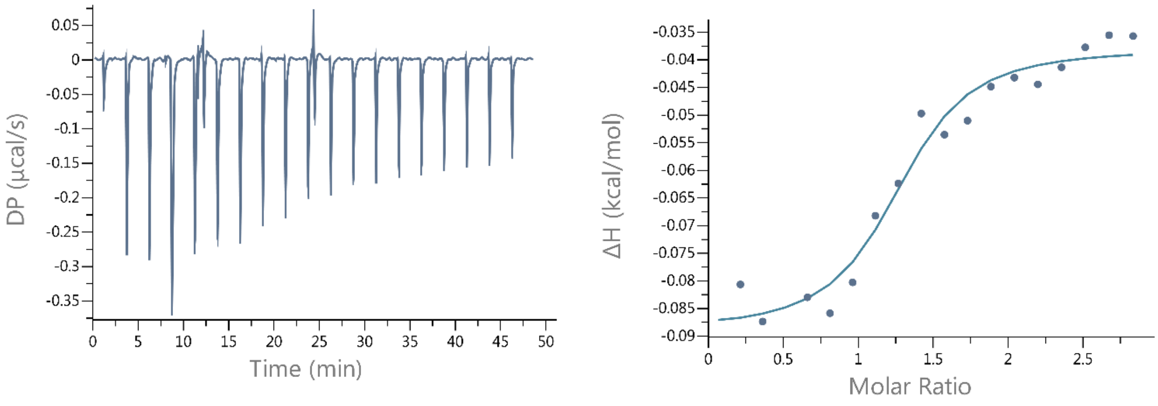

H-ʟ-Lys-OH to receptor 1:

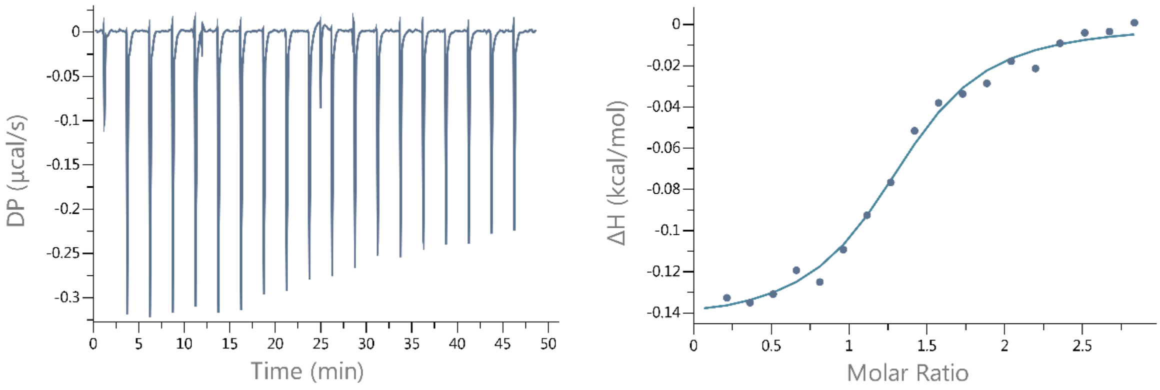

H-ᴅ-Trp-OH to receptor 1:

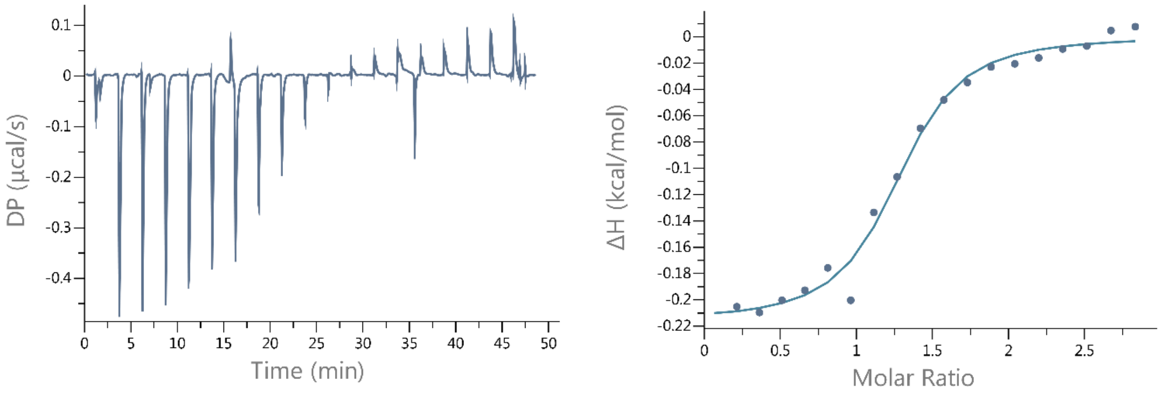

Peptide 2 to receptor 1:

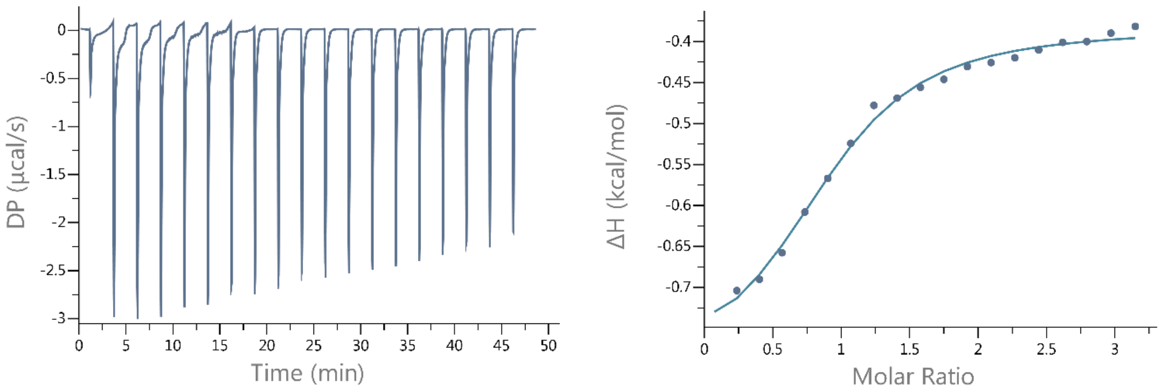

Peptide 3 to receptor 1:

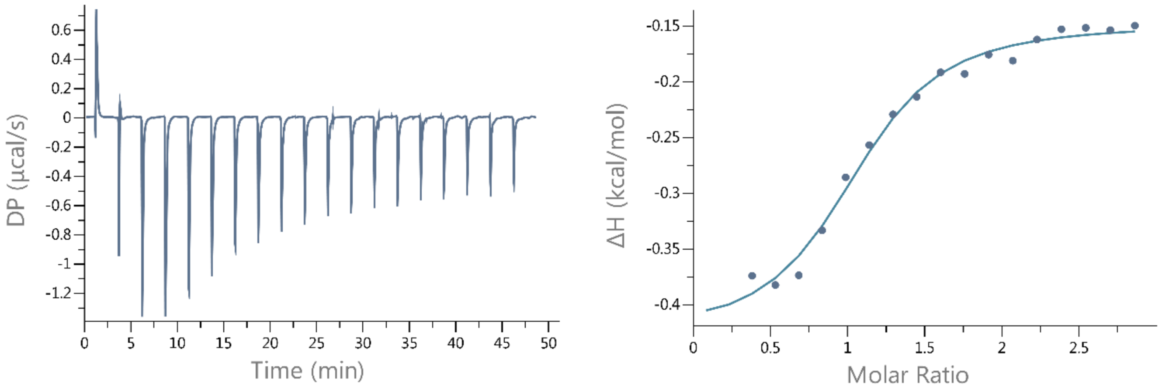

Peptide 4 to receptor 1:

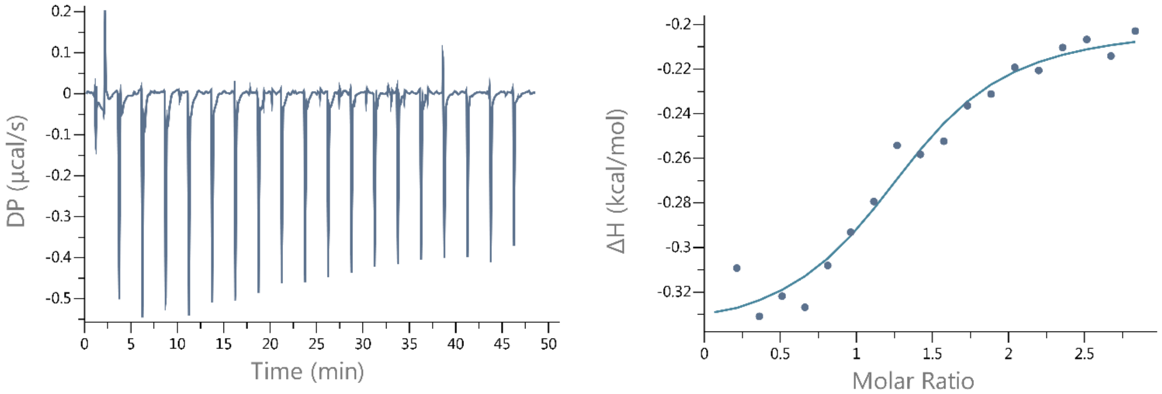

Peptide 5 to receptor 1:

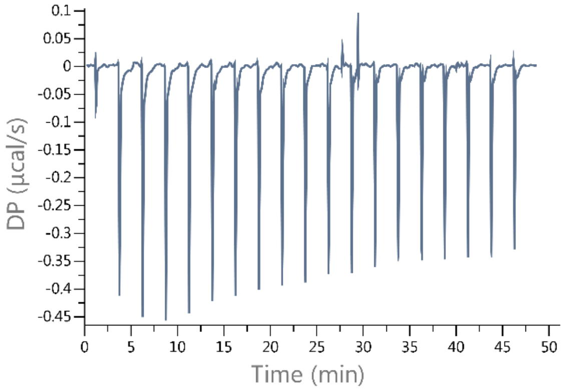

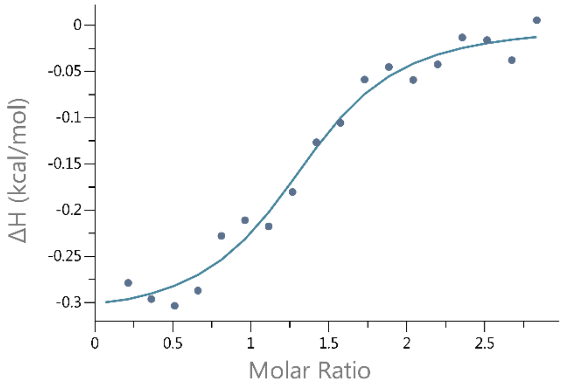

Peptide 6 to receptor 1:

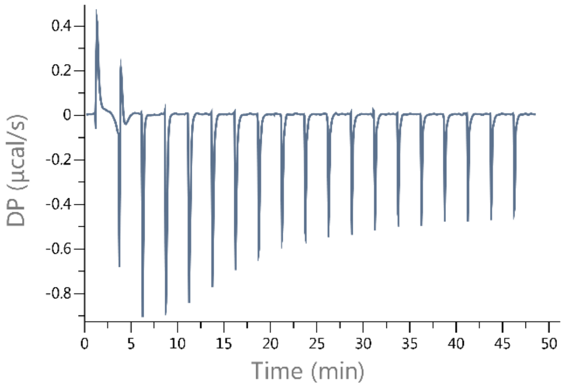

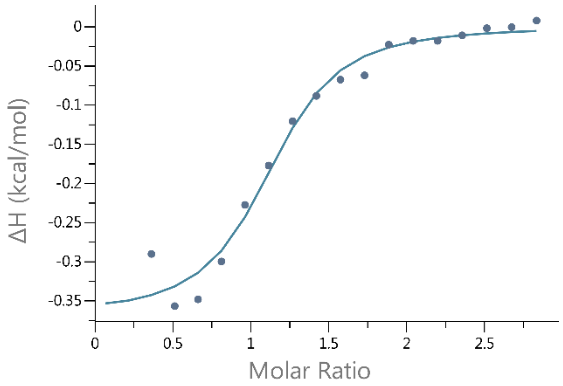

Peptide 7 to receptor 1:

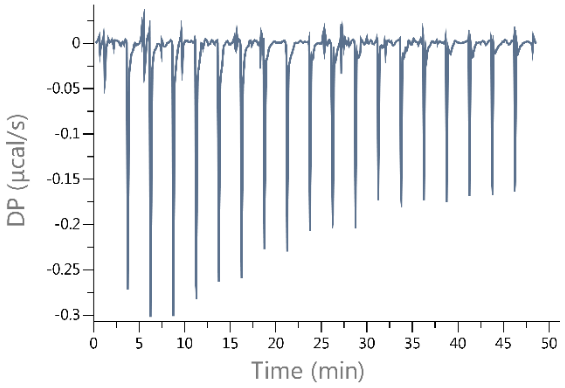

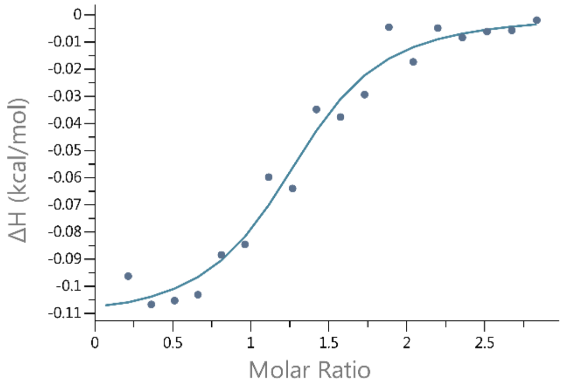

Peptide 8 to receptor 1:

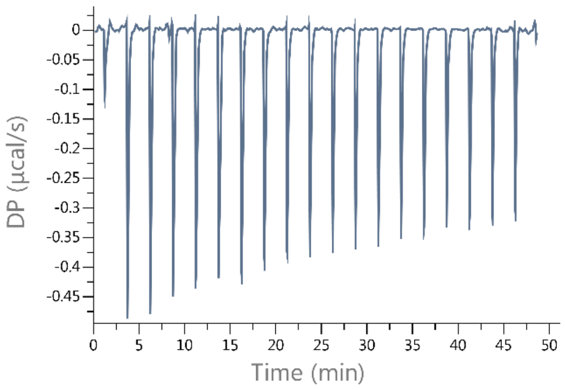

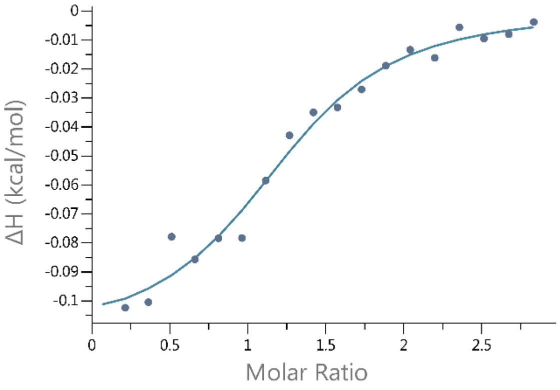

4.2 Fluorescence titrations.

The titration experiments were performed in an 0.5 mL cuvette in milli-Q H_2O at room temperature. The concentration of the host solution was 25 μM and the starting volume 0.5 mL. Two guest solutions of 12.5 mM and 125 μM concentrations in milli-Q H_2O were used and aliquots of these solution were added to reach the final concentration of 5 mM (the only exceptions were the titrations with Leu and Glu as guests where higher solution concentrations of 50 mM and 250 mM were used to achieve a detectable change in the emission spectrum of the receptor upon guest titration). The excitation wavelength was 235 nm. Changes in the intensity in the emission spectrum of receptor **1** over a wavelength range of λ = 320-341 nm were monitored. The obtained data was then fitted to a 1:1 stoichiometry model using the online BindFit software (supramolecular.org) to obtain K_a values of the complexes.

Injection table:

Titration	Amount of guest solution 1 added (μL)	Amount of guest solution 2 added (μL)	Equivalents of guest added	Concentration of guest (mM)
1	0	-	0	0
2	1	-	1	0.025
3	1	-	2	0.05
4	1	-	3	0.075
5	1	-	4	0.01
6	2	-	6	0.015
7	2		8	0.2
8	-	0.5	13	0.325
9	-	0.7	20	0.5
10	-	1.0	30	0.75
11	-	1.0	40	1.0
12	-	2.0	60	1.5
13	-	4.0	100	2.5
14	-	10.0	200	5.0

H-ʟ-Trp-OH to receptor 1:

Fluorescence emission spectra of **1** upon addition of increasing portions of guest:

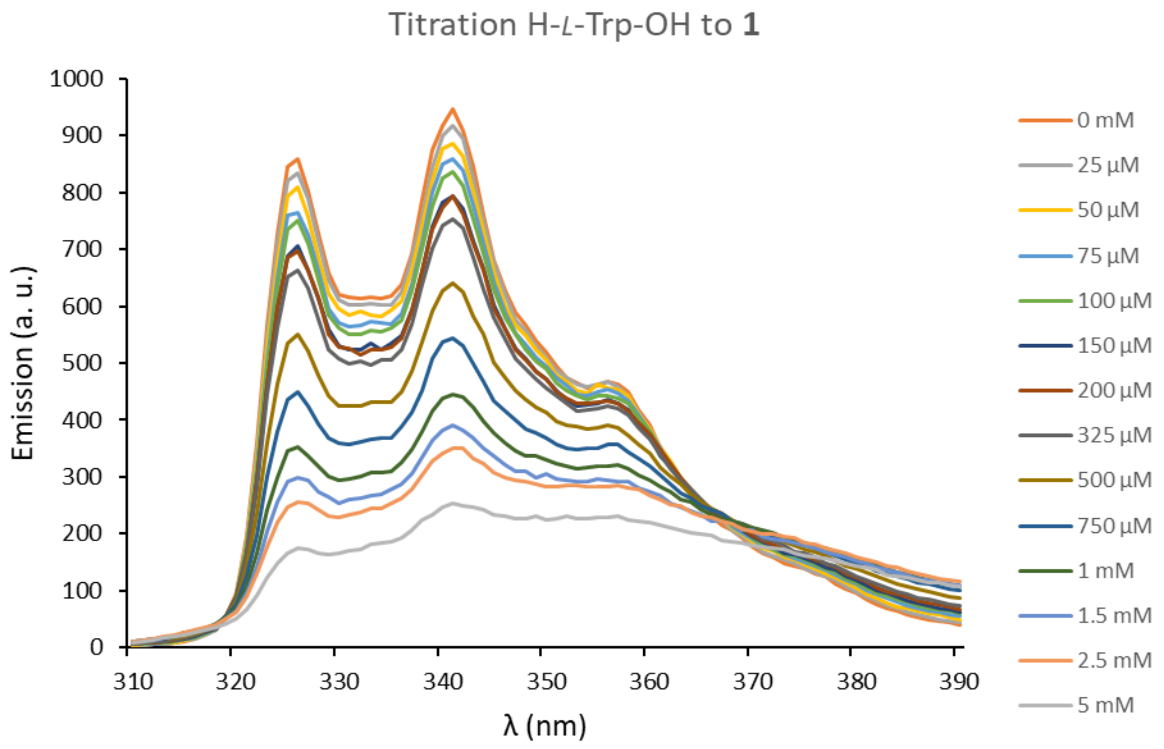

Titration curve (5 selected wavelengths from the 320–341 nm range shown for clarity):

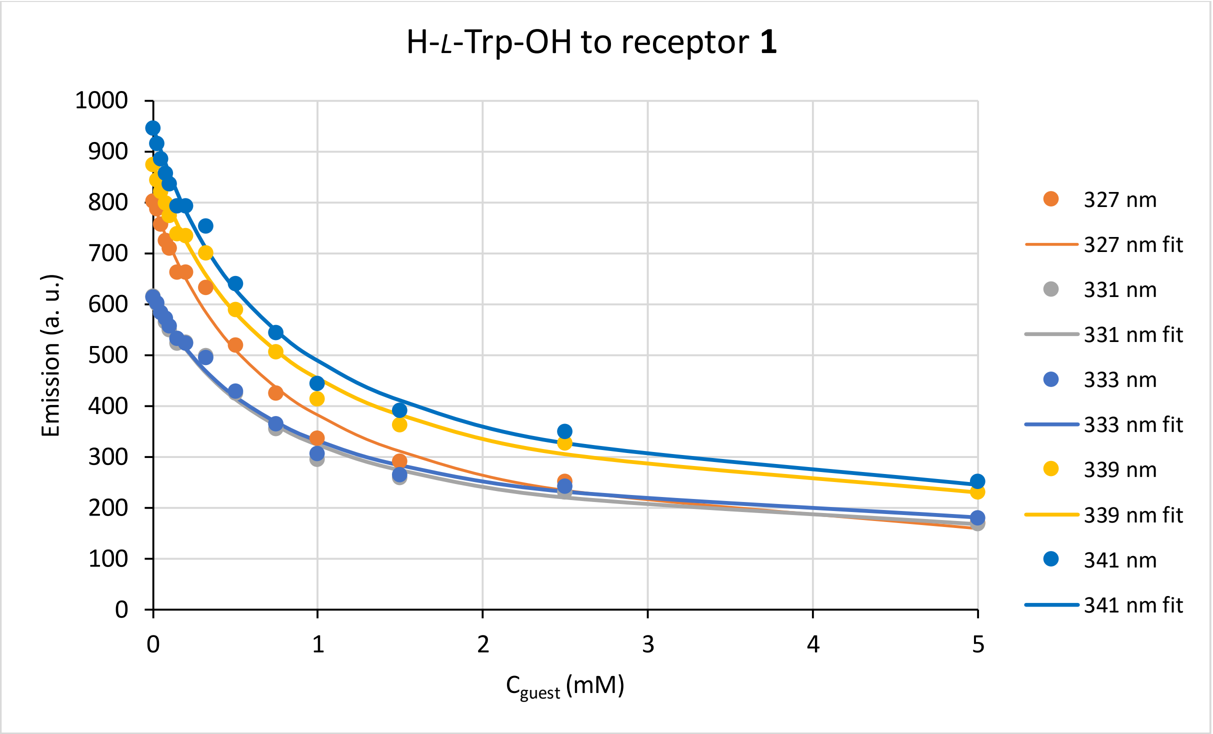

K_a value obtained by fitting the data for the wavelength range λ = 320-341 nm: $\boldsymbol{K_a}$ **= 1338 ± 27 M**$^{-1}$

Link to the fitted data: http://app.supramolecular.org/bindfit/view/b71daadb-1d40-40b6-9fa0-89db4396a324

H-L-Leu-OH to receptor 1:

Fluorescence emission spectra of **1** upon addition of increasing portions of guest:

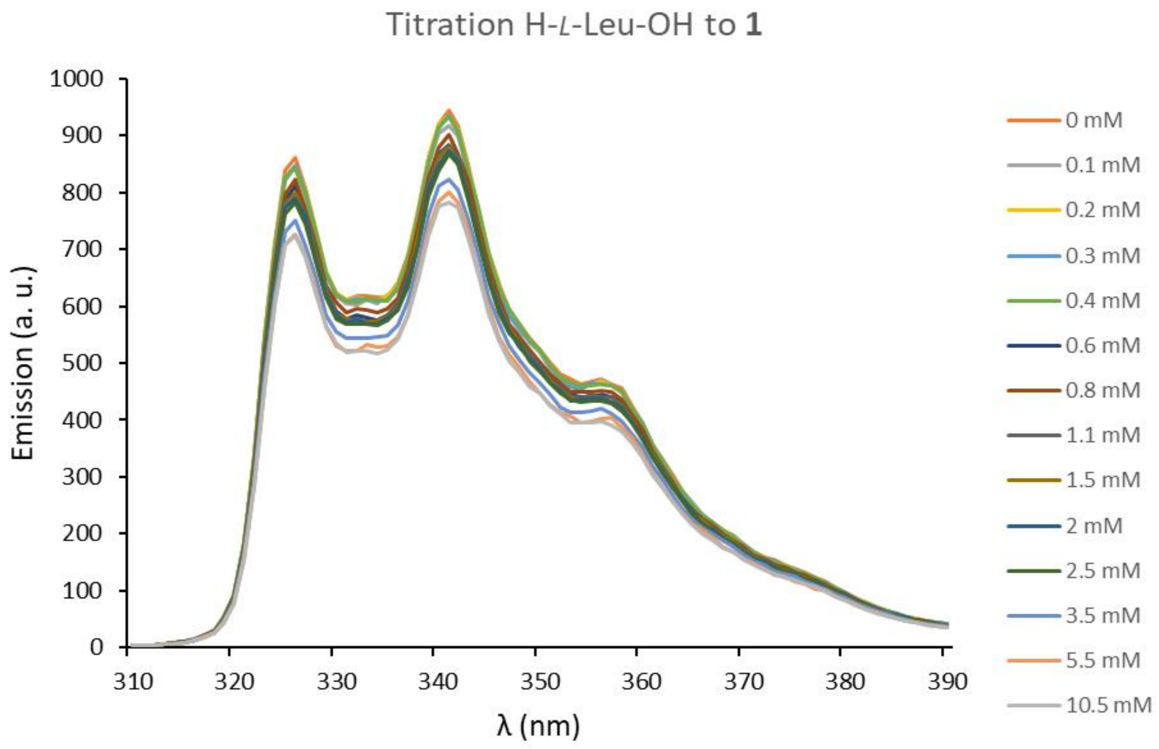

Titration curve (5 selected wavelengths from the 320–341 nm range shown for clarity):

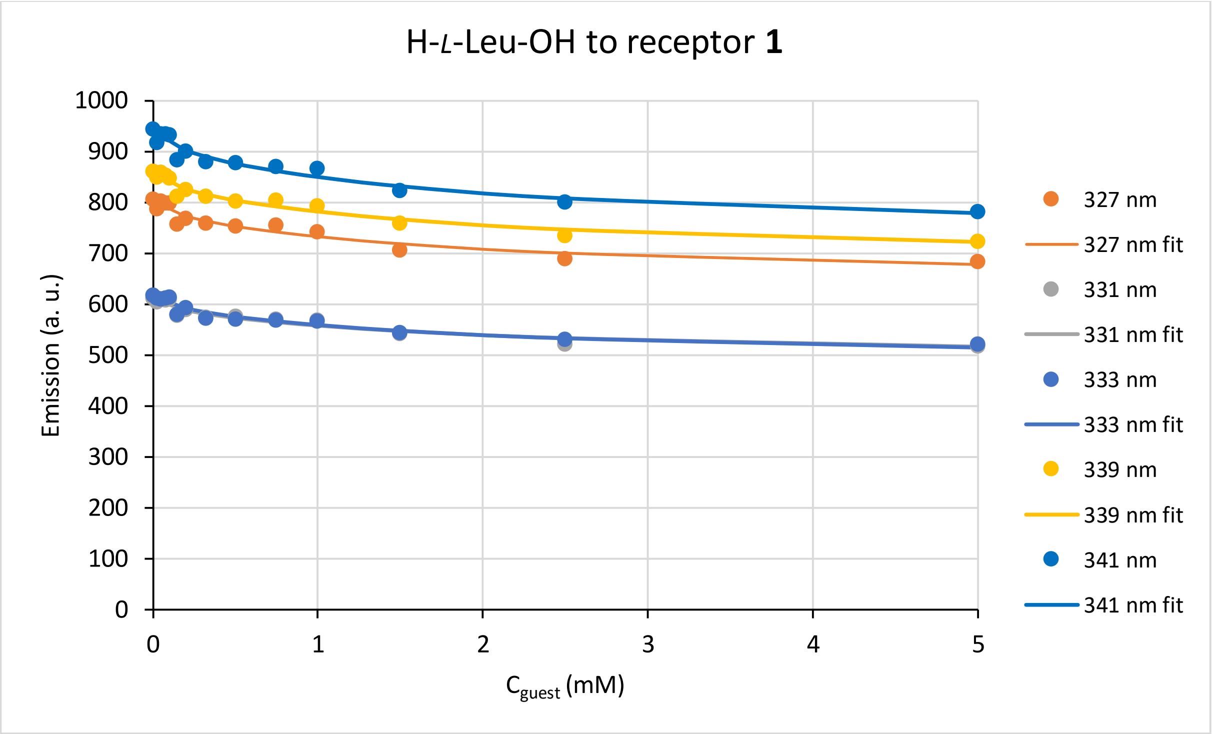

K_a value obtained by fitting the data for the wavelength range λ = 320-341 nm: $\boldsymbol{K_a}$ **= 328 ± 12 M^{-1}**

Link to the fitted data: http://app.supramolecular.org/bindfit/view/d44bc1d9-c9c4-4ad0-9b27-5d1997671dad

H-ʟ-Phe-OH to receptor 1:

Fluorescence emission spectra of **1** upon addition of increasing portions of guest:

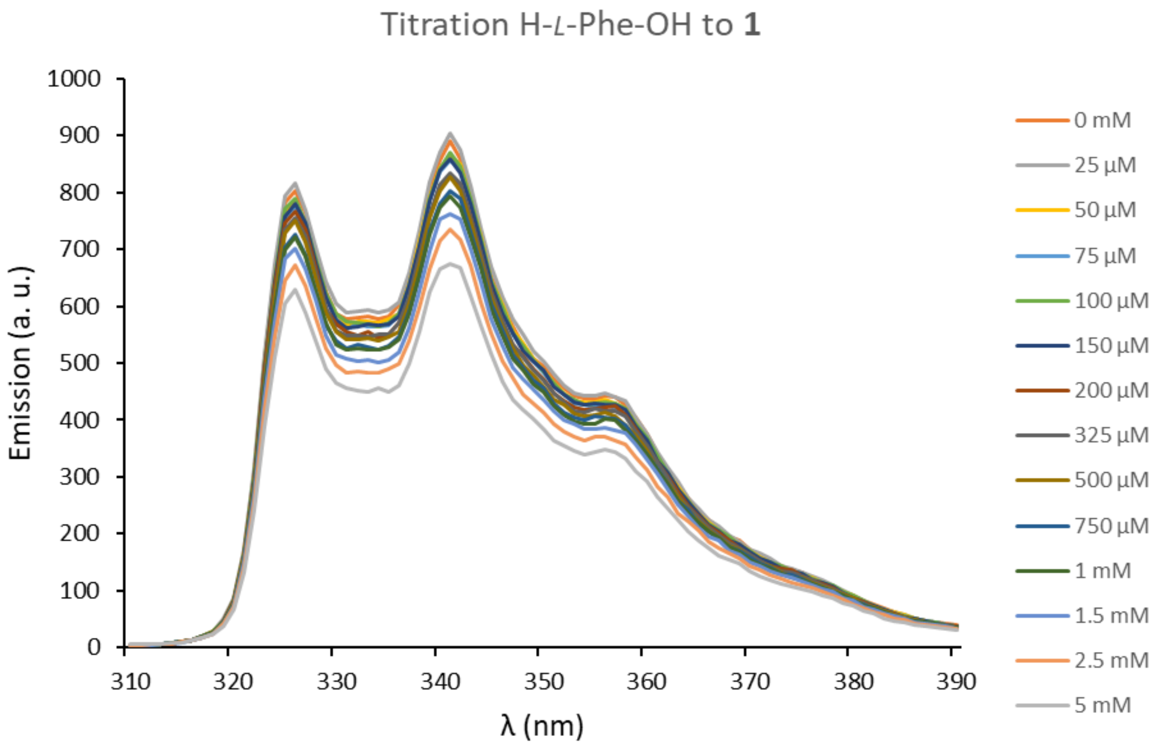

Titration curve (5 selected wavelengths from the 320–341 nm range shown for clarity):

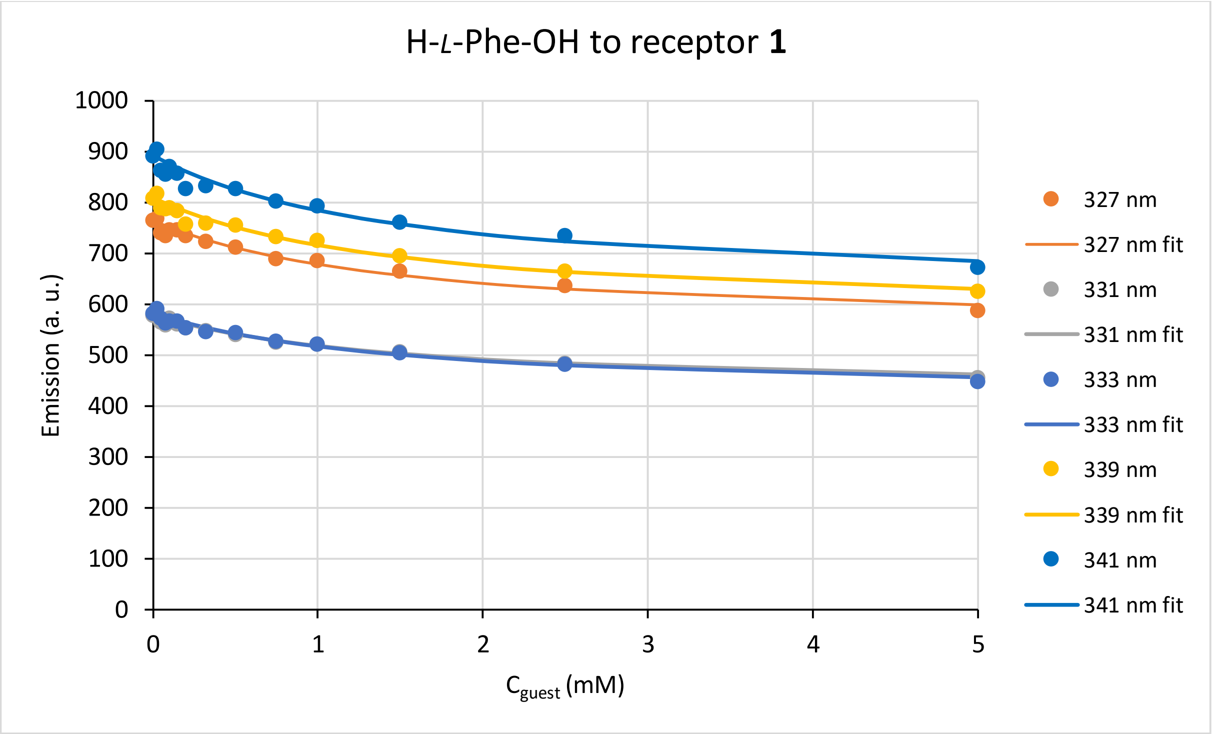

K_a value obtained by fitting the data for the wavelength range λ = 320-341 nm: $\boldsymbol{K_a}$ **= 624 ± 19 M**$^{-1}$

Link to the fitted data: http://app.supramolecular.org/bindfit/view/5f3fd5ad-f1c1-4bcf-8d47-a1581da1e3bf

H-ʟ-Glu-OH to receptor 1:

Fluorescence emission spectra of **1** upon addition of increasing portions of guest:

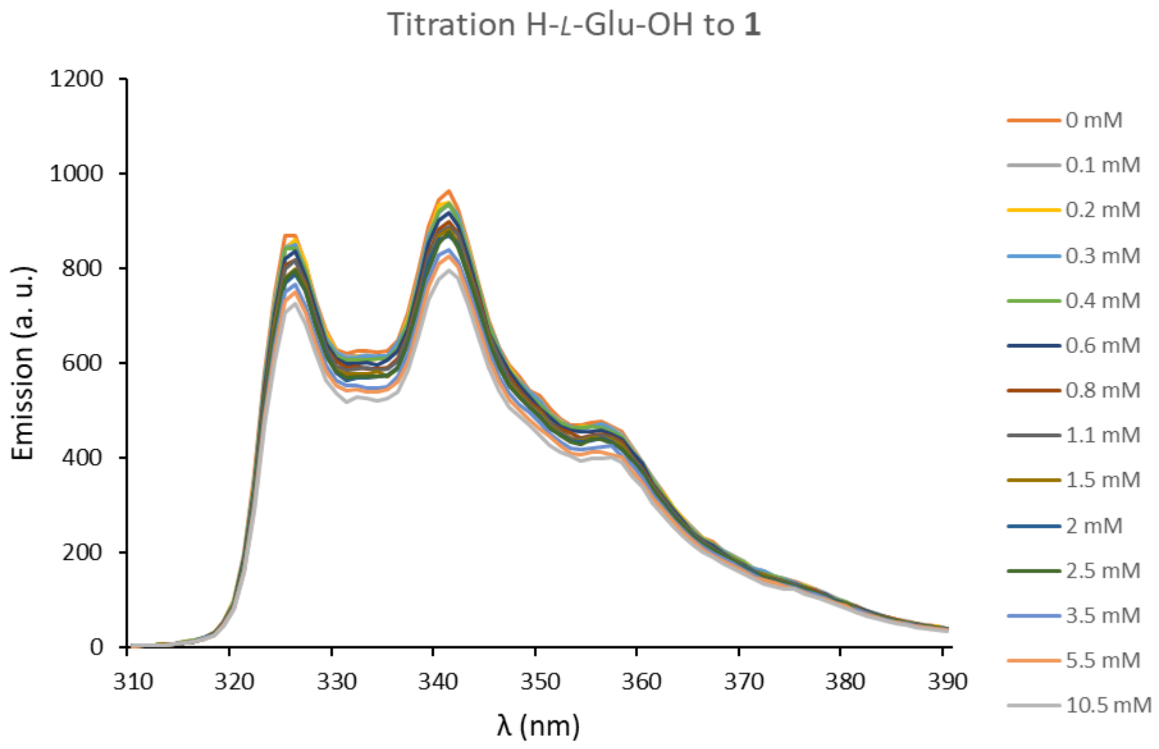

Titration curve (5 selected wavelengths from the 320–341 nm range shown for clarity):

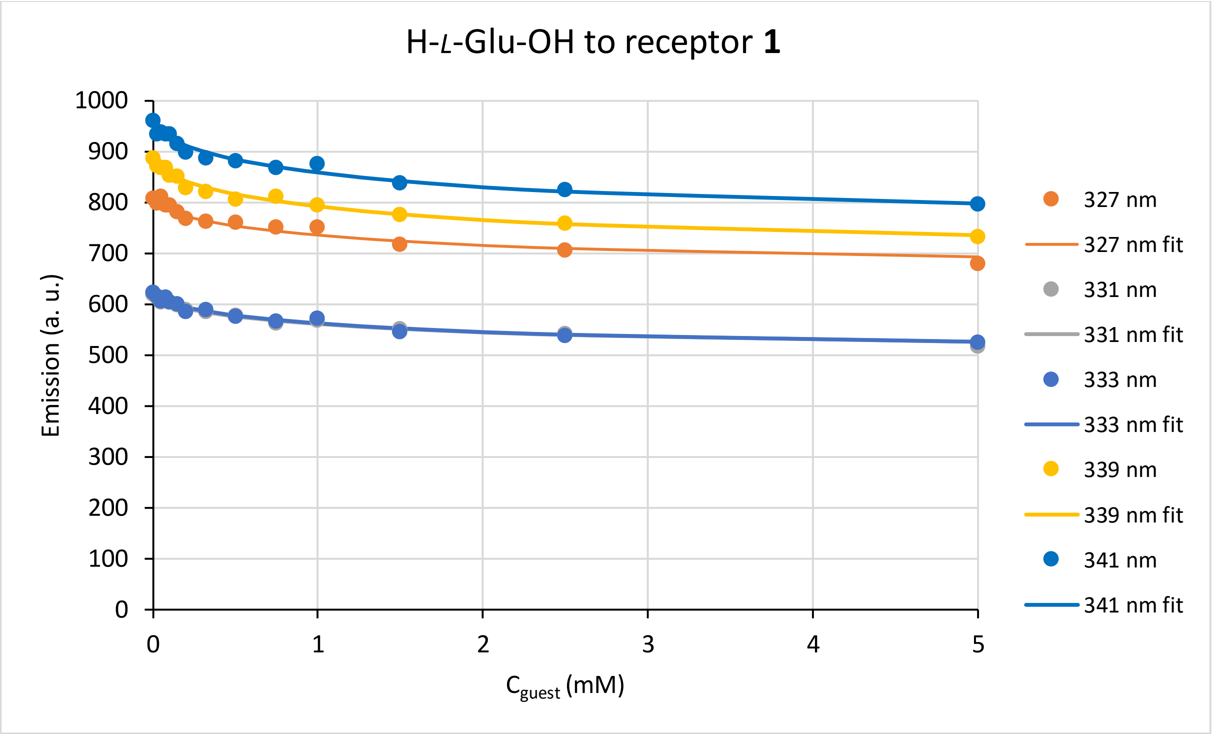

K_a value obtained by fitting the data for the wavelength range λ = 320-341 nm: $\boldsymbol{K_a}$ **= 463 ± 12 M**$^{-1}$

Link to the fitted data: http://app.supramolecular.org/bindfit/view/0bf996c7-415f-4612-9814-28797d843a63

H-ʟ-Lys-OH to receptor 1:

Fluorescence emission spectra of **1** upon addition of increasing portions of guest:

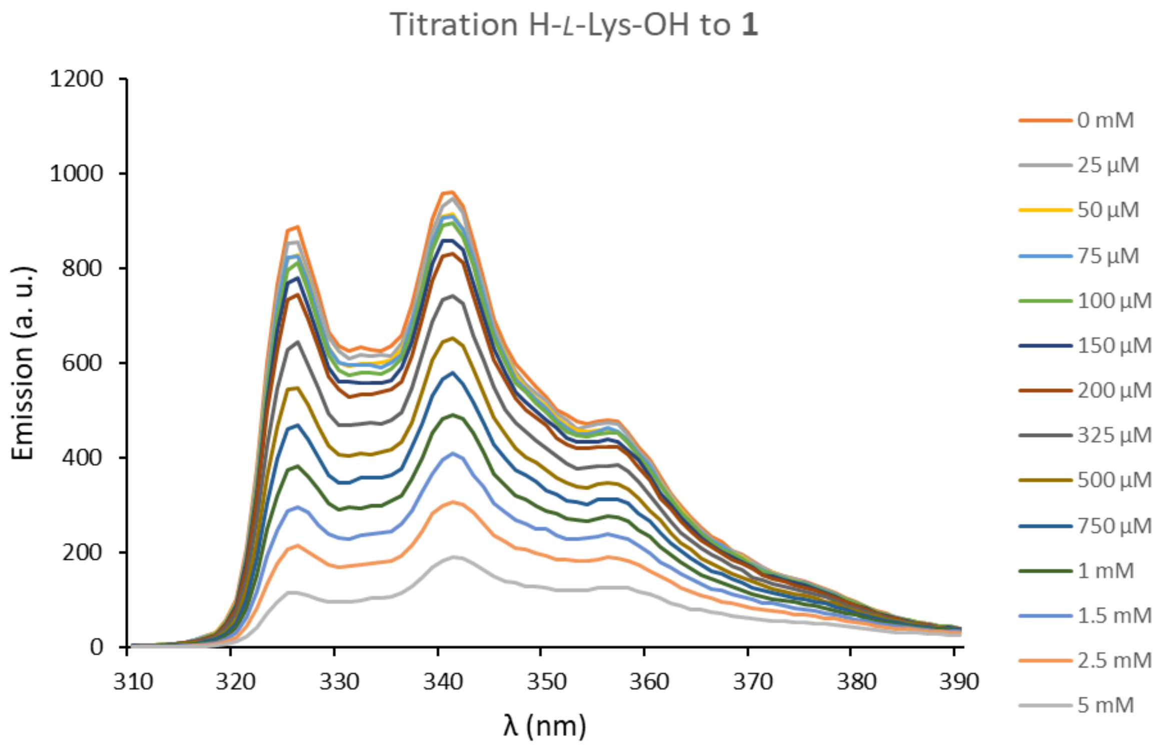

Titration curve (5 selected wavelengths from the 320–341 nm range shown for clarity):

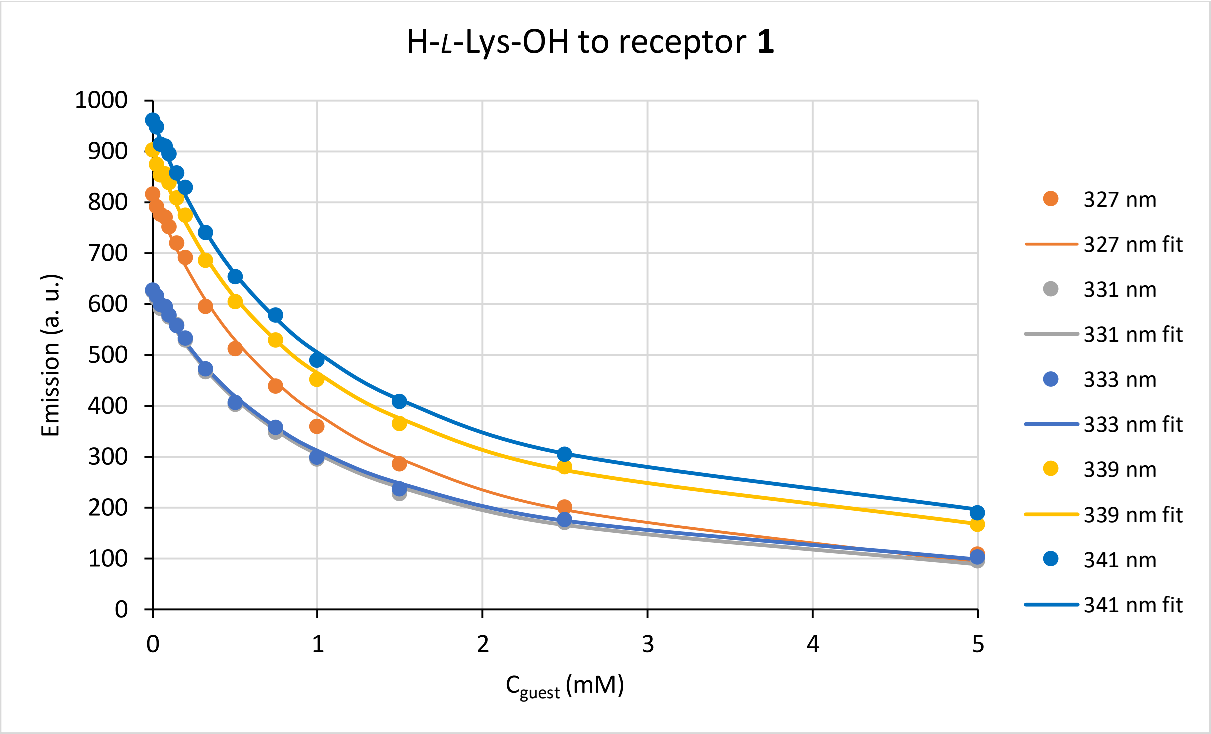

K_a value obtained by fitting the data for the wavelength range λ = 320-341 nm: $\boldsymbol{K_a}$ **= 1059 ± 12 M**$^{-1}$

Link to the fitted data: http://app.supramolecular.org/bindfit/view/c7211284-73c3-4016-8c3b-eab9d9a1d156

H-ᴅ-Trp-OH to receptor 1:

Fluorescence emission spectra of **1** upon addition of increasing portions of guest:

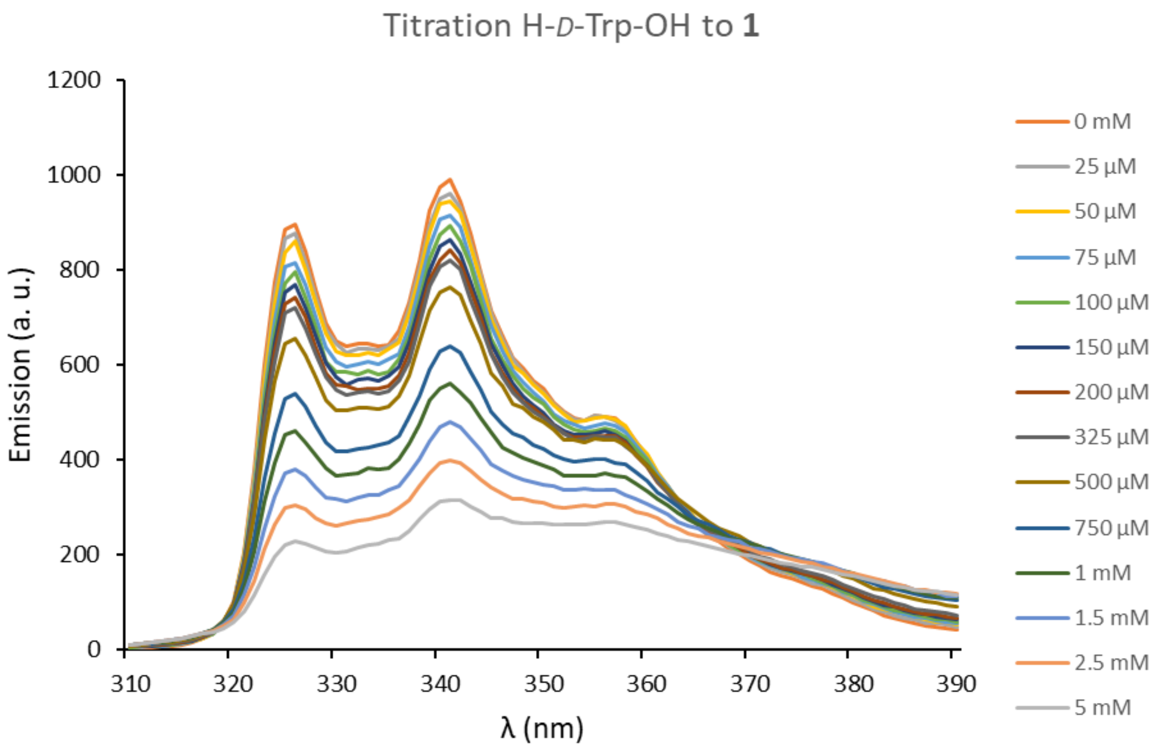

Titration curve (5 selected wavelengths from the 320–341 nm range shown for clarity):

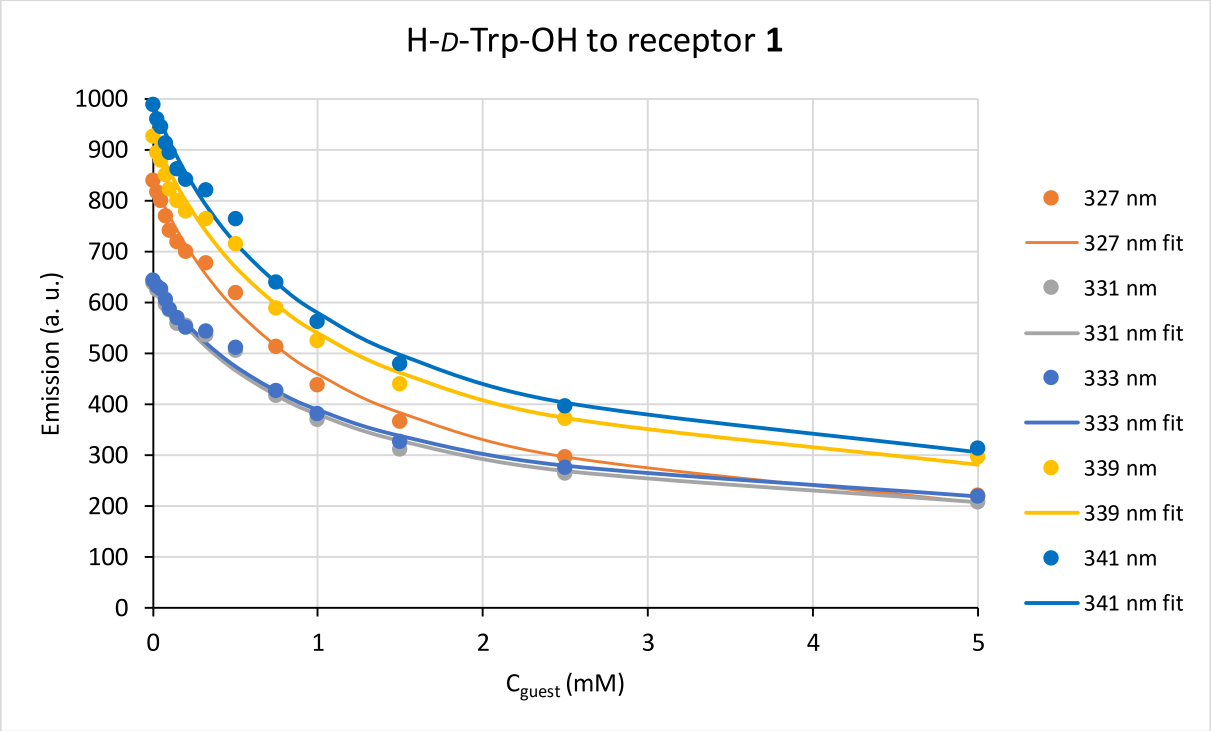

K_a value obtained by fitting the data for the wavelength range λ = 320-341 nm: $\boldsymbol{K_a}$ **= 1027 ± 20 M**$^{-1}$

Link to the fitted data: http://app.supramolecular.org/bindfit/view/ca0a709c-036d-48d7-b342-71c29c73c50a

H-TrpAlaAla-NH$_2$ to receptor 1:

Fluorescence emission spectra of **1** upon addition of increasing portions of guest:

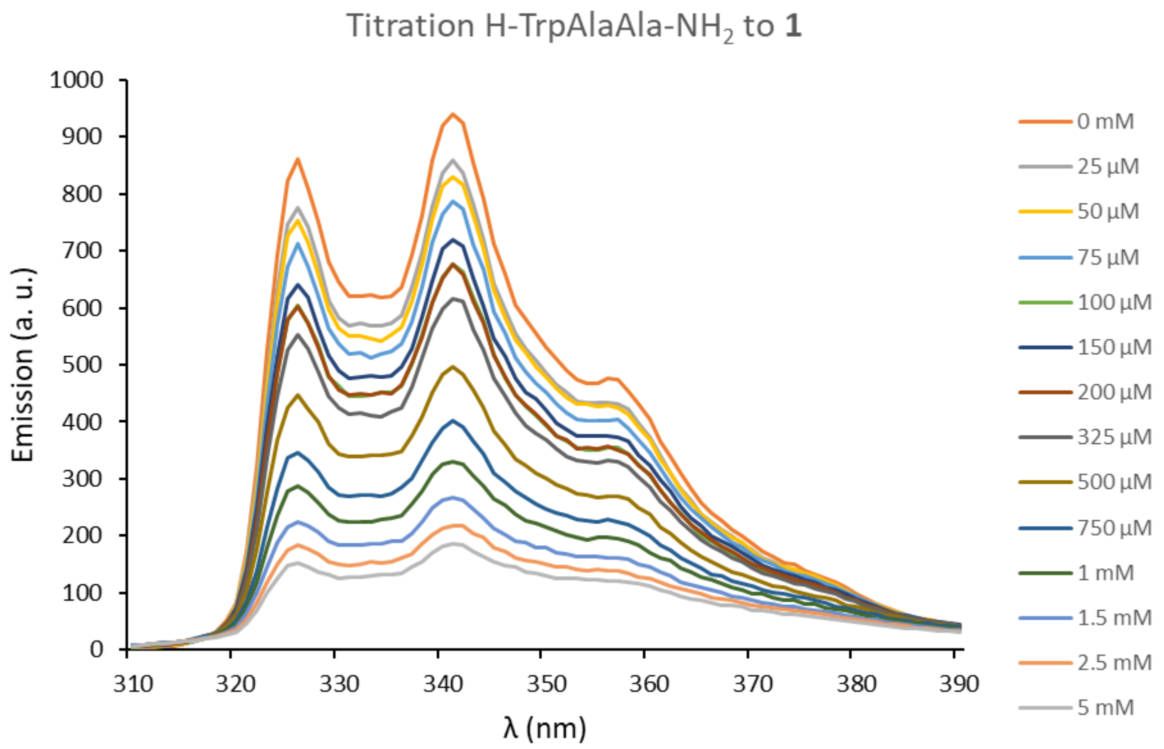

Titration curve (5 selected wavelengths from the 320–341 nm range shown for clarity):

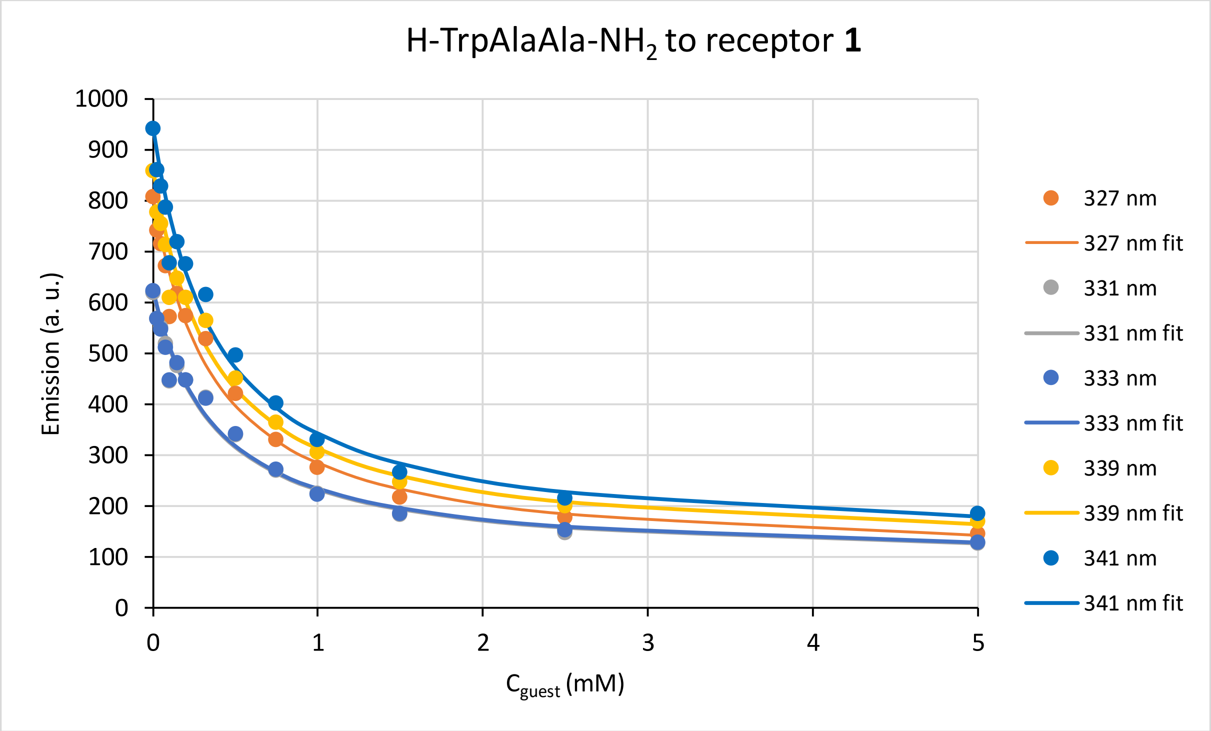

K_a value obtained by fitting the data for the wavelength range λ = 320-341 nm: $\boldsymbol{K_a}$ **= 2817 ± 85 M^{-1}**

Link to the fitted data: http://app.supramolecular.org/bindfit/view/5dd26231-c0bd-44a0-aafa-8133913ba30f

H-AlaTrpAla-NH_2 to receptor 1:

Fluorescence emission spectra of **1** upon addition of increasing portions of guest:

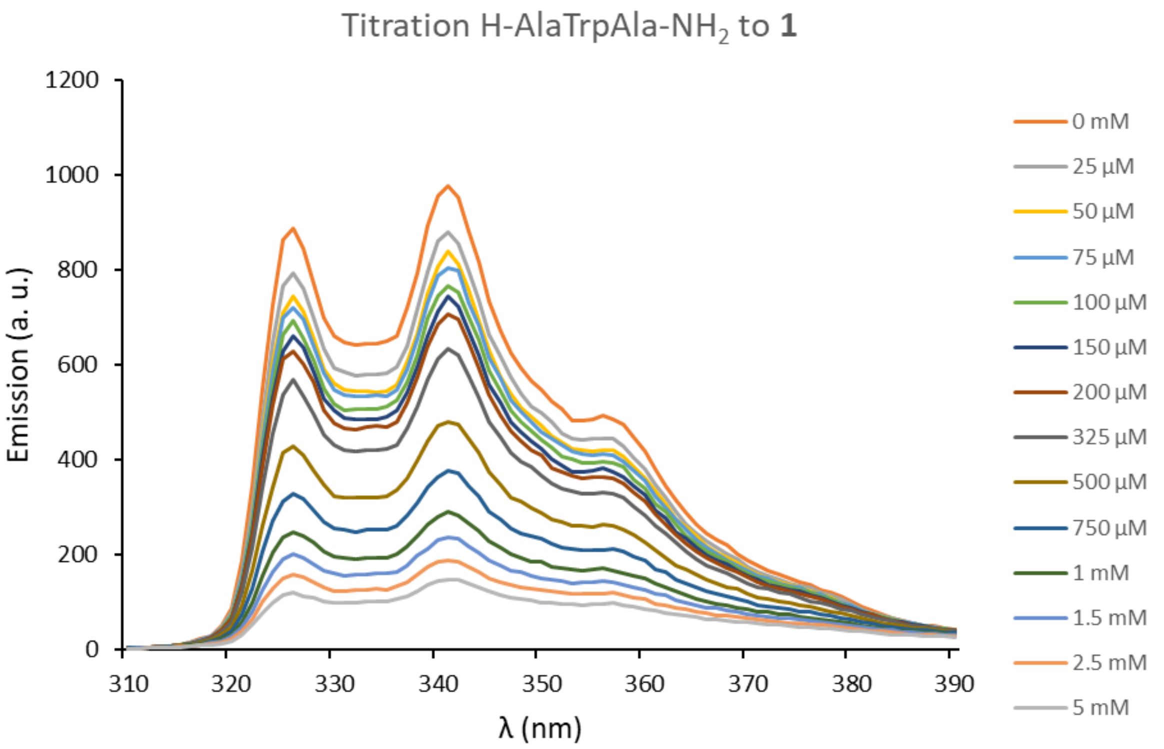

Titration curve (5 selected wavelengths from the 320–341 nm range shown for clarity):

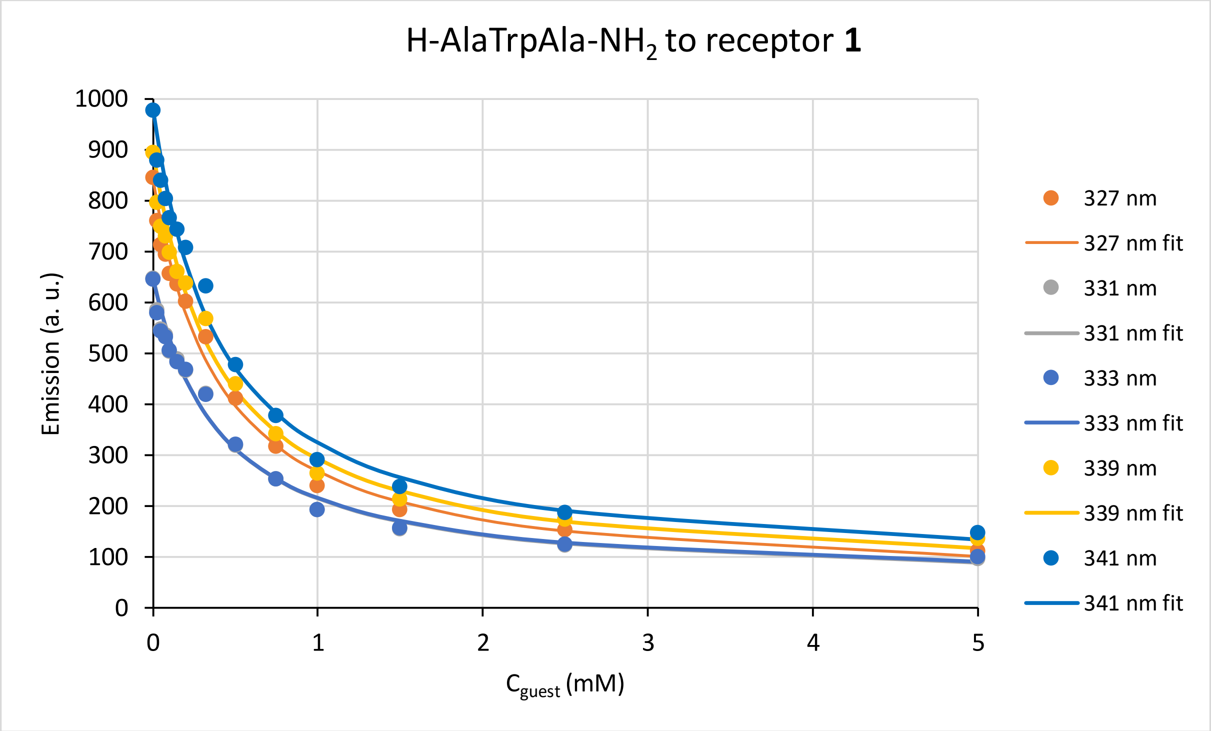

K_a value obtained by fitting the data for the wavelength range λ = 320-341 nm: $\boldsymbol{K_a}$ **= 2627 ± 66 M^{-1}**

Link to the fitted data: http://app.supramolecular.org/bindfit/view/d1c29a5b-e669-4994-915e-0929c51a33cd

H-AlaAlaTrp-NH_2 to receptor 1:

Fluorescence emission spectra of **1** upon addition of increasing portions of guest:

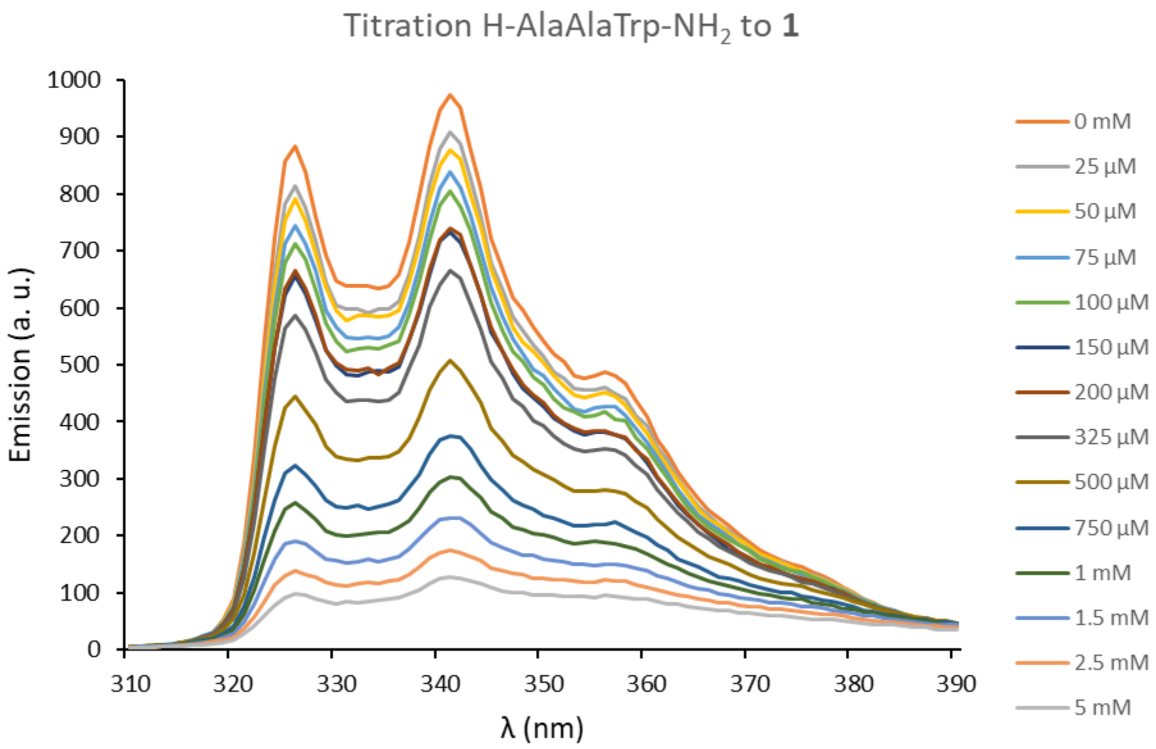

Titration curve (5 selected wavelengths from the 320–341 nm range shown for clarity):

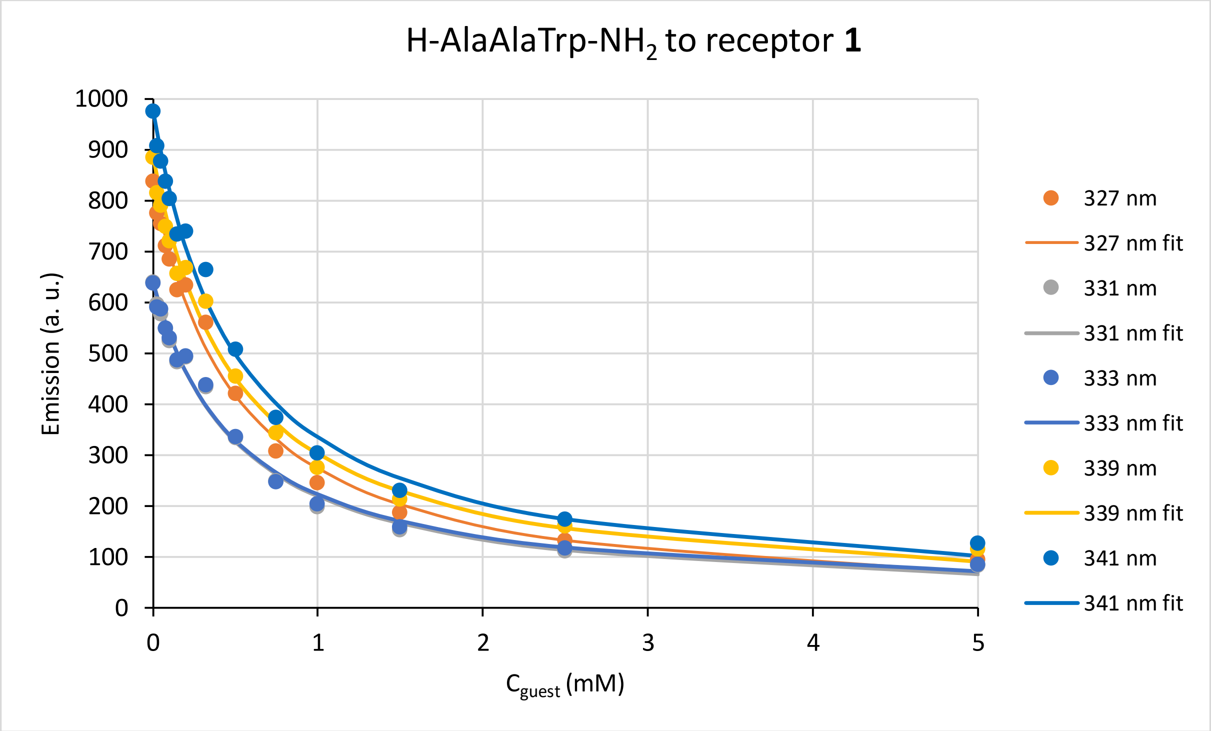

K_a value obtained by fitting the data for the wavelength range λ = 320-341 nm: $\boldsymbol{K_a}$ **= 2058 ± 41 M^{-1}**

Link to the fitted data: http://app.supramolecular.org/bindfit/view/2e5d7eb7-d0fb-48d8-8bbb-c630bdbb76ac

Ac-TrpAlaAla-NH_2 to receptor 1:

Fluorescence emission spectra of **1** upon addition of increasing portions of guest:

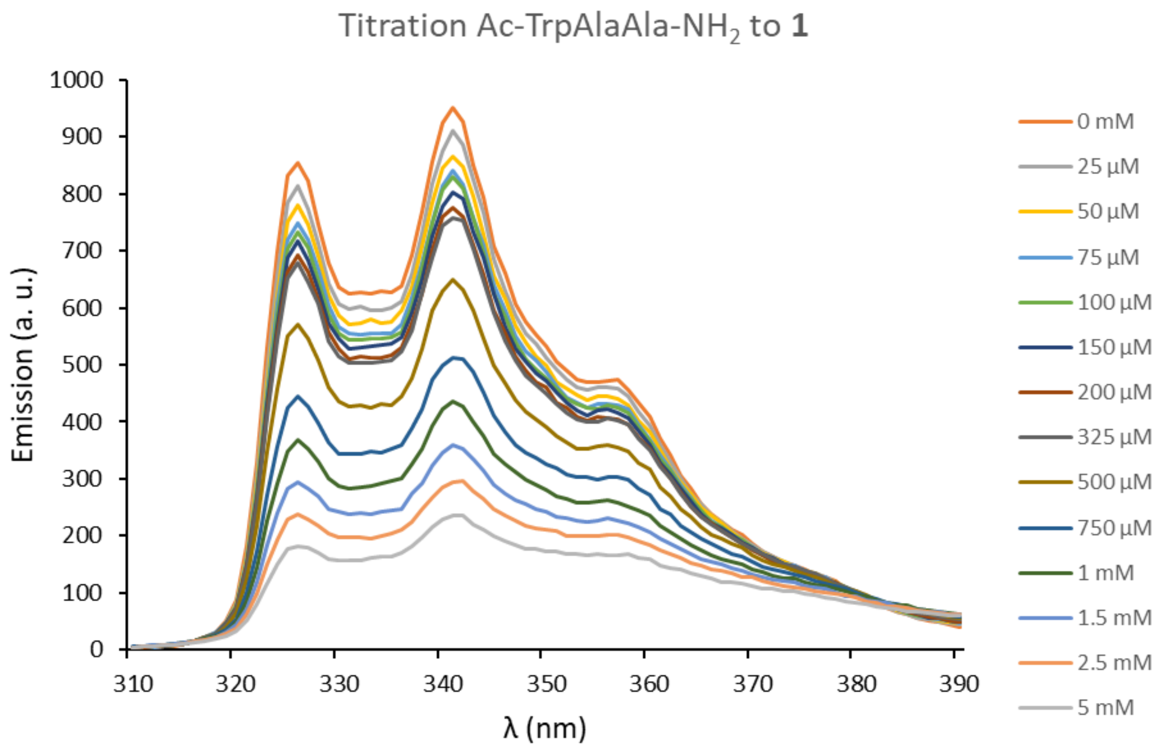

Titration curve (5 selected wavelengths from the 320–341 nm range shown for clarity):

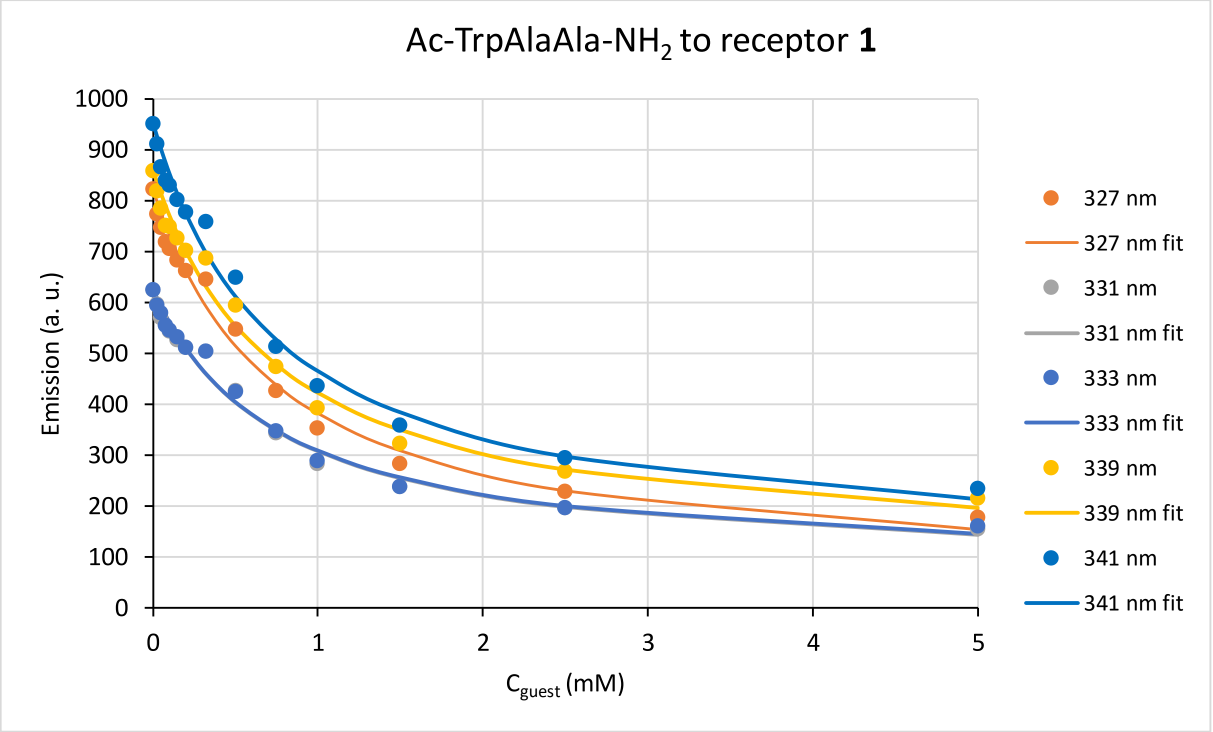

K_a value obtained by fitting the data for the wavelength range λ = 320-341 nm: $\boldsymbol{K_a}$ **= 1378 ± 56 M^{-1}**

Link to the fitted data: http://app.supramolecular.org/bindfit/view/73ad1b39-cbe4-41c6-bc2e-ef0b94879c74

Ac-AlaTrpAla-NH$_2$ to receptor 1:

Fluorescence emission spectra of **1** upon addition of increasing portions of guest:

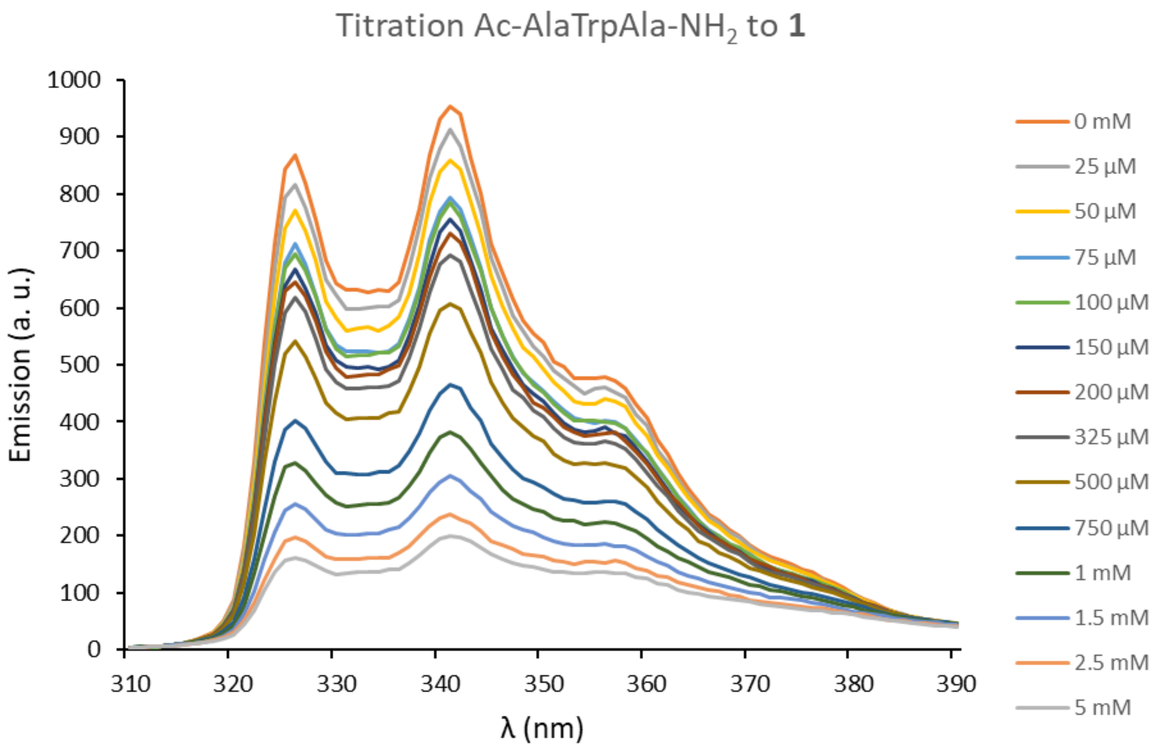

Titration curve (5 selected wavelengths from the 320–341 nm range shown for clarity):

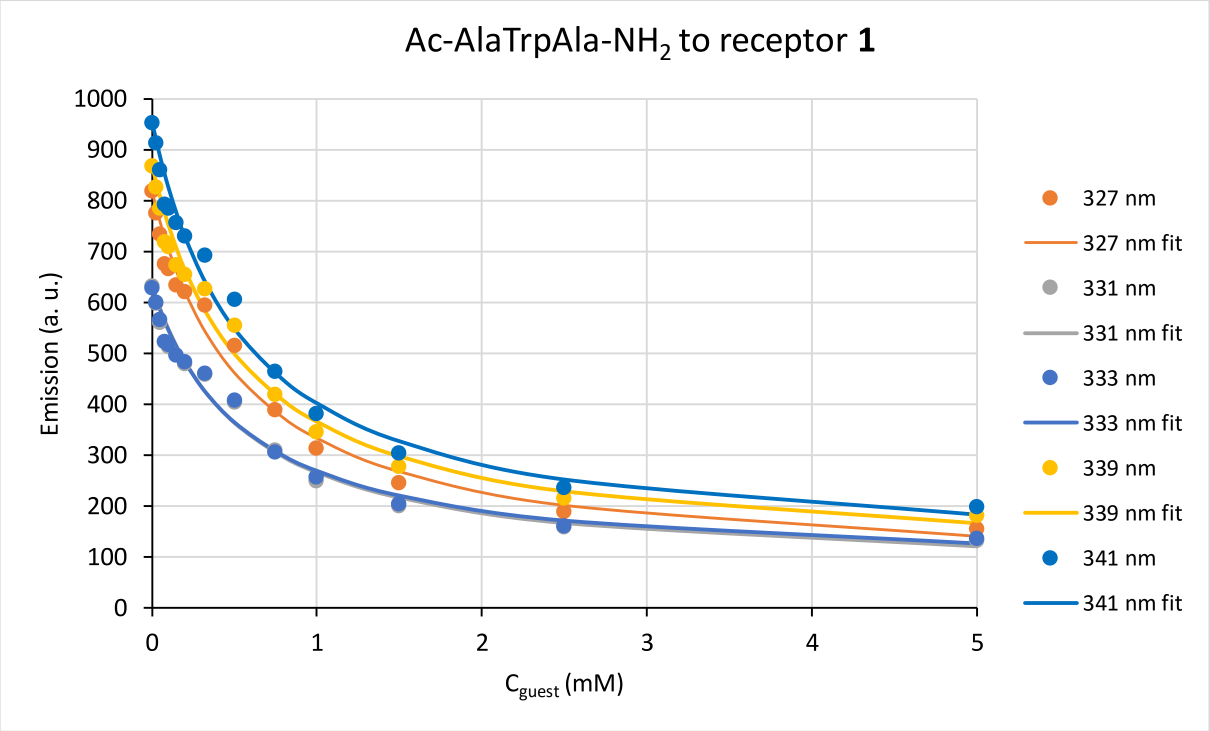

K_a value obtained by fitting the data for the wavelength range λ = 320-341 nm: $\boldsymbol{K_a}$ **= 1910 ± 55 M^{-1}**

Link to the fitted data: http://app.supramolecular.org/bindfit/view/70cea03b-d898-4855-83eb-06c5a043bc2e

Ac-AlaAlaTrp-NH$_2$ to receptor 1:

Fluorescence emission spectra of **1** upon addition of increasing portions of guest:

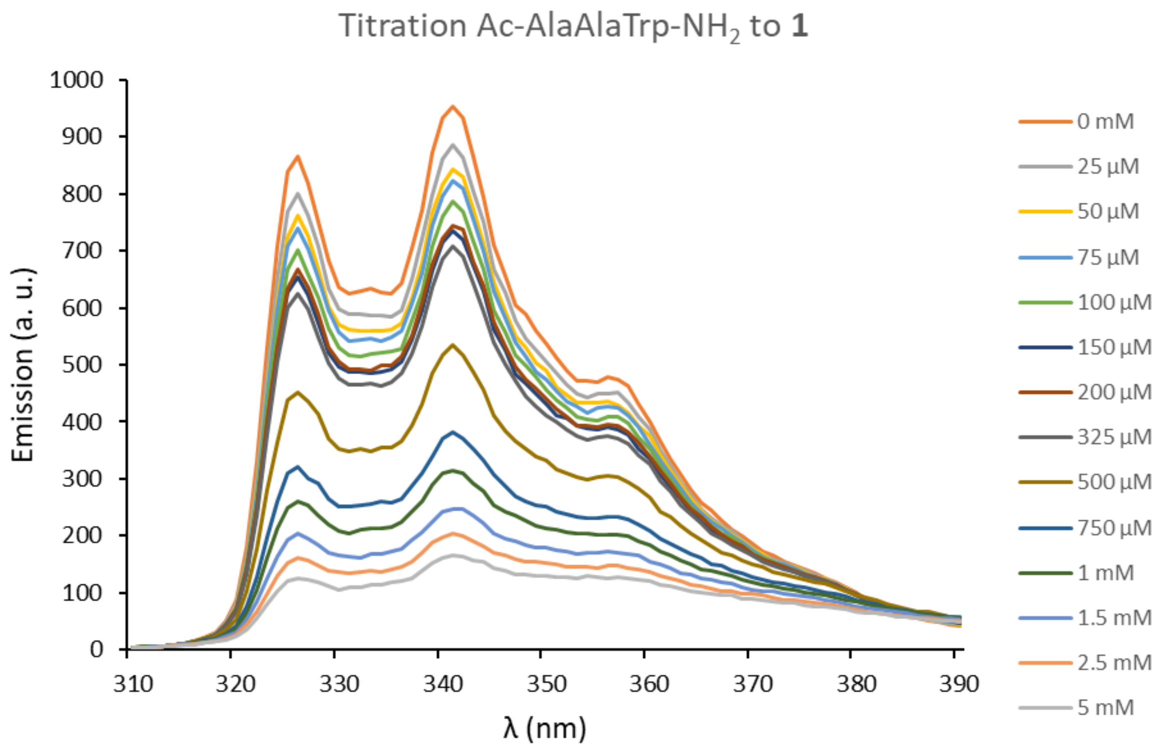

Titration curve (5 selected wavelengths from the 320–341 nm range shown for clarity):

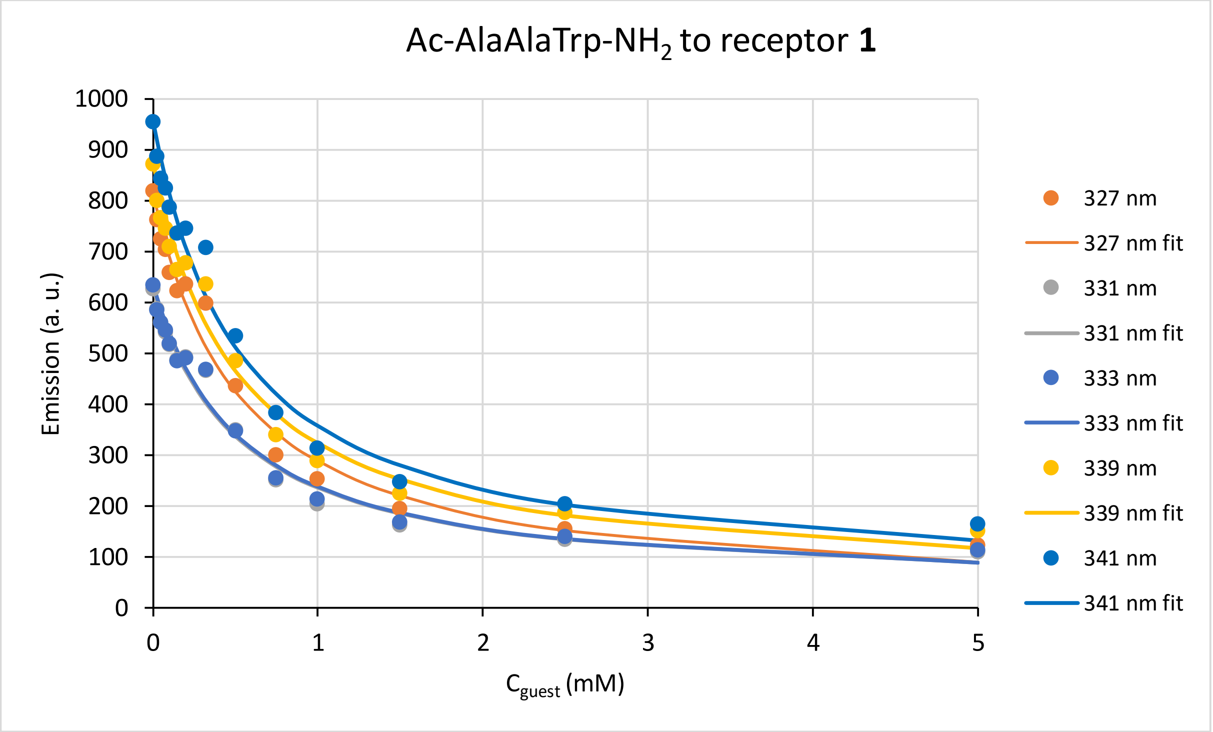

K_a value obtained by fitting the data for the wavelength range λ = 320-341 nm: $\boldsymbol{K_a}$ **= 1978 ± 59 M^{-1}**

Link to the fitted data: http://app.supramolecular.org/bindfit/view/e8bdbf5a-ca95-4ddc-89d3-30117a0560e1

H-AlaAlaAla-NH_2 to receptor 1:

Fluorescence emission spectra of **1** upon addition of increasing portions of guest:

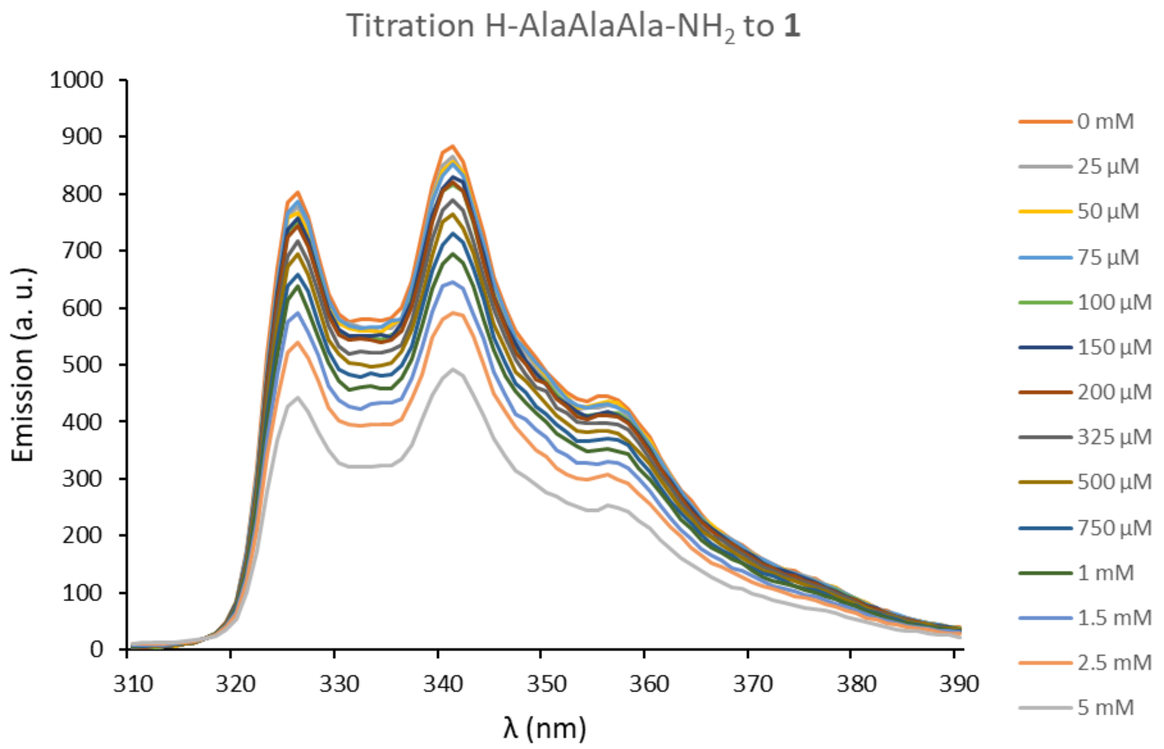

Titration curve (5 selected wavelengths from the 320–341 nm range shown for clarity):

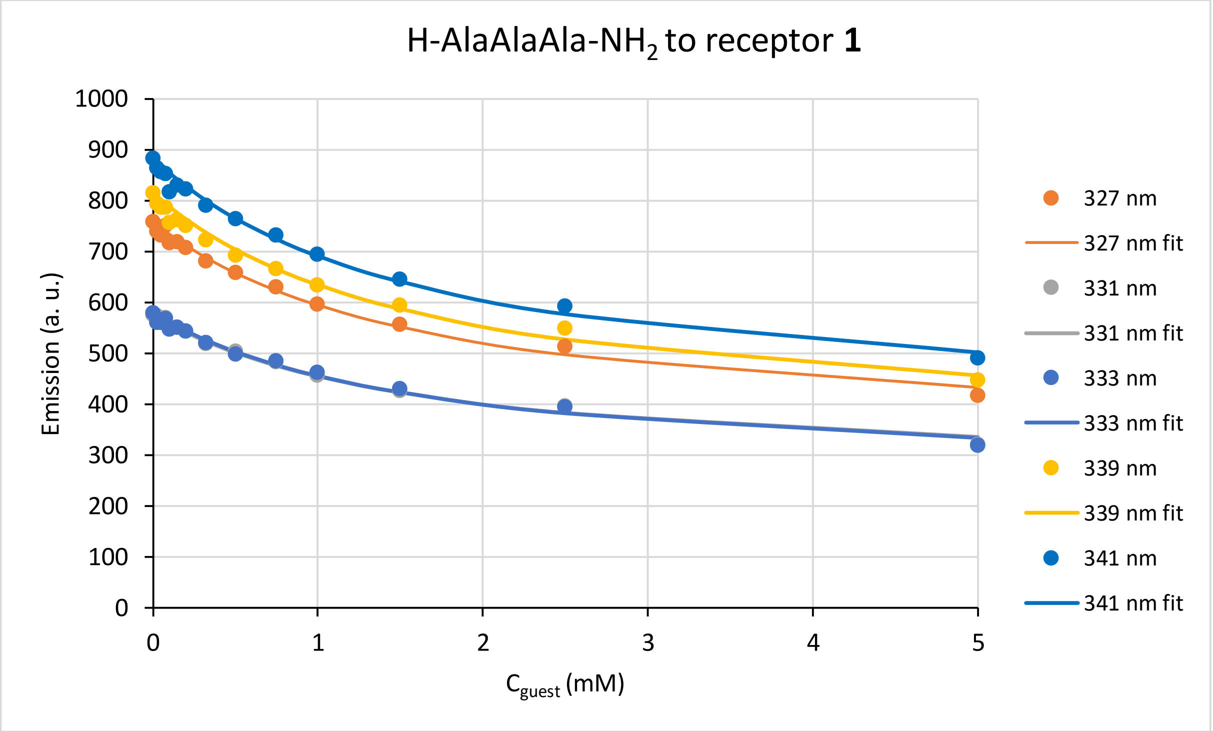

K_a value obtained by fitting the data for the wavelength range λ = 320-341 nm: $\boldsymbol{K_a}$ **= 637 ± 14 M^{-1}**

Link to the fitted data: http://app.supramolecular.org/bindfit/view/031bb90d-ef0c-49fd-b0d6-c9330d5bcf55

5. NMR spectra.

^{1}H NMR spectrum – peptide **2** (CD_3OD, 400 MHz)

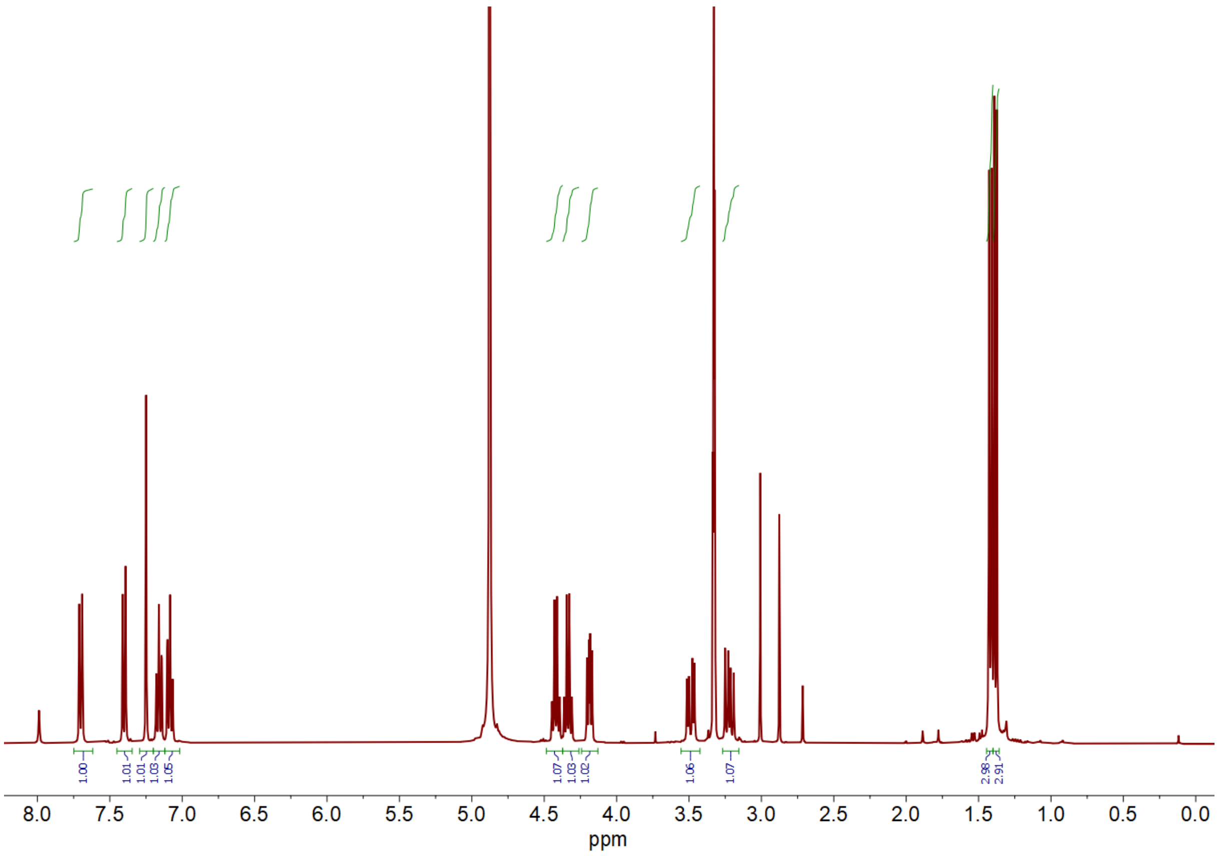

^{13}C NMR spectrum – peptide **2** (CD_3OD, 101 MHz)

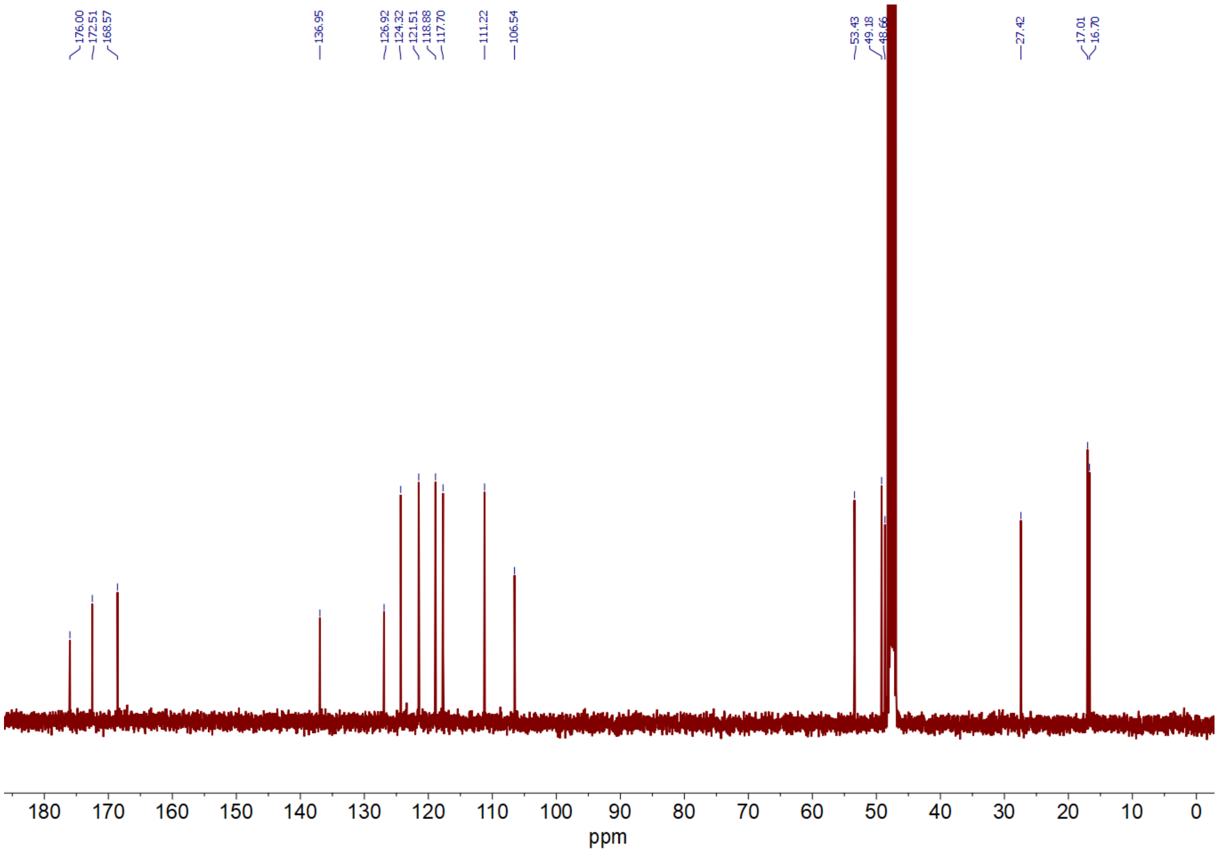

^{1}H NMR spectrum – peptide **3** (CD_3OD, 400 MHz)

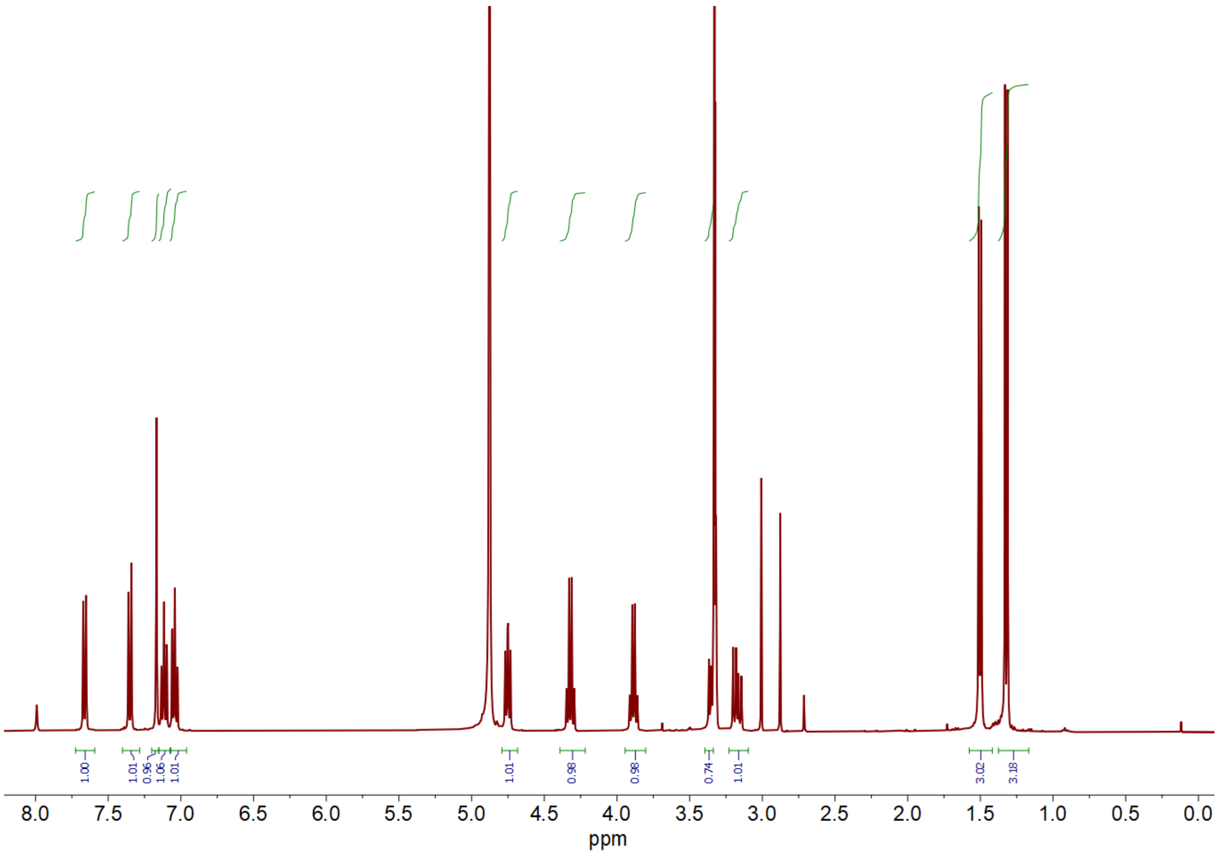

^{13}C NMR spectrum – peptide **3** (CD_3OD, 101 MHz)

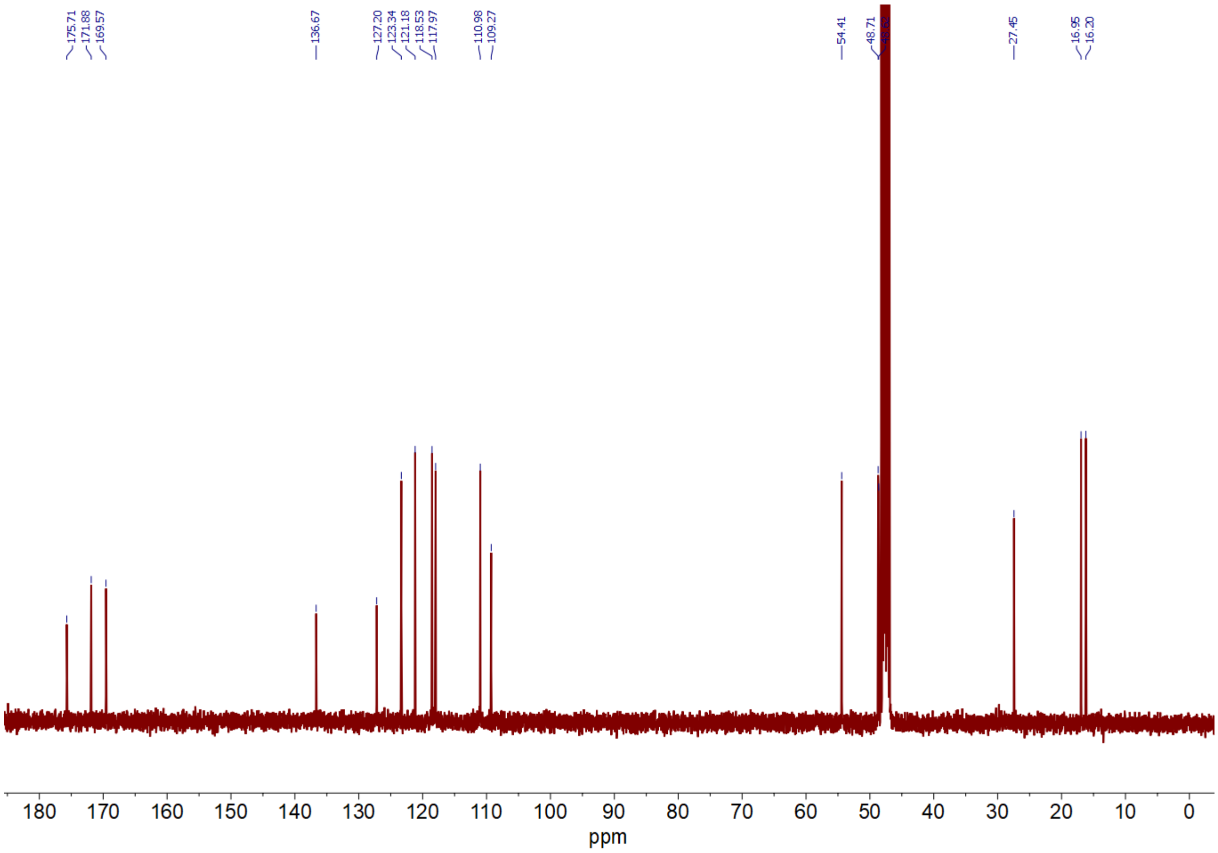

^{1}H NMR spectrum – peptide **4** (CD_3OD, 400 MHz)

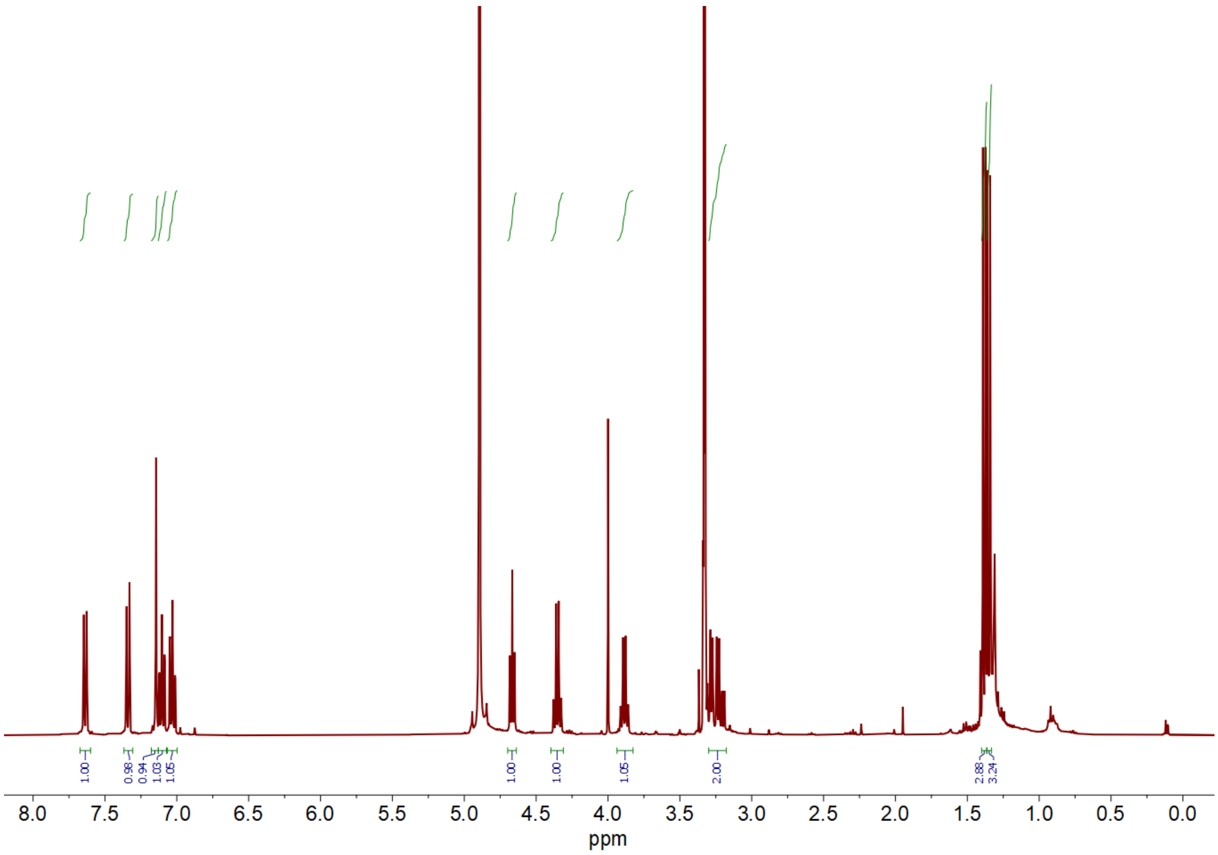

^{13}C NMR spectrum – peptide **4** (CD_3OD, 101 MHz)

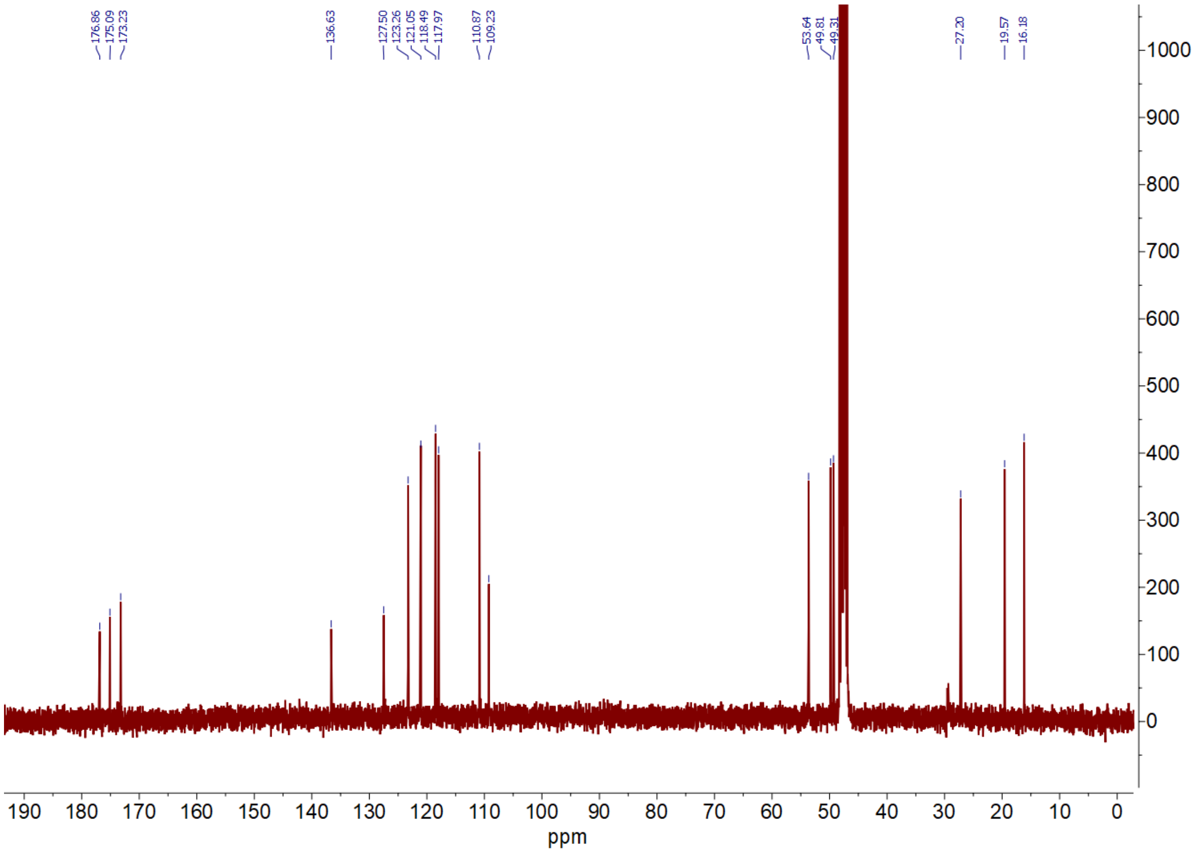

^{1}H NMR spectrum – peptide **5** (CD_3OD, 400 MHz)

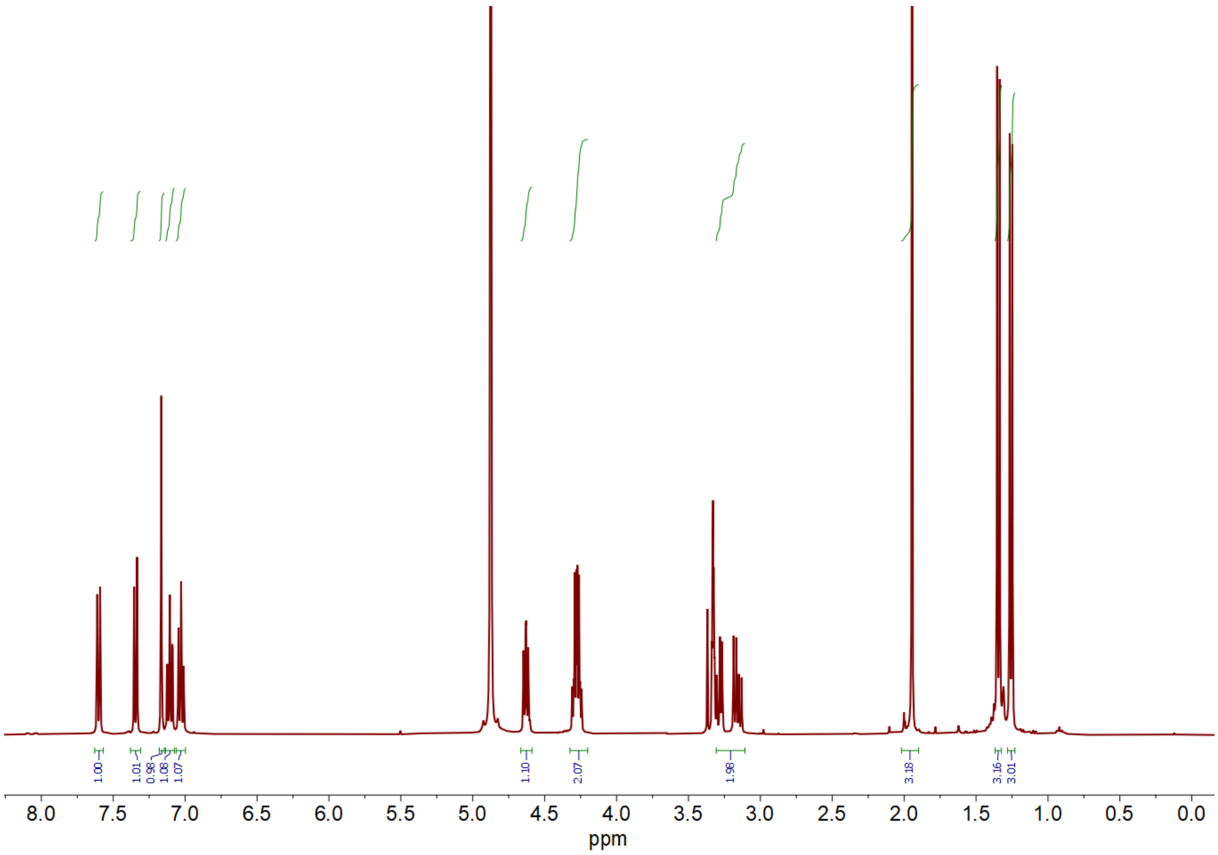

^{13}C NMR spectrum – peptide **5** (CD_3OD, 101 MHz)

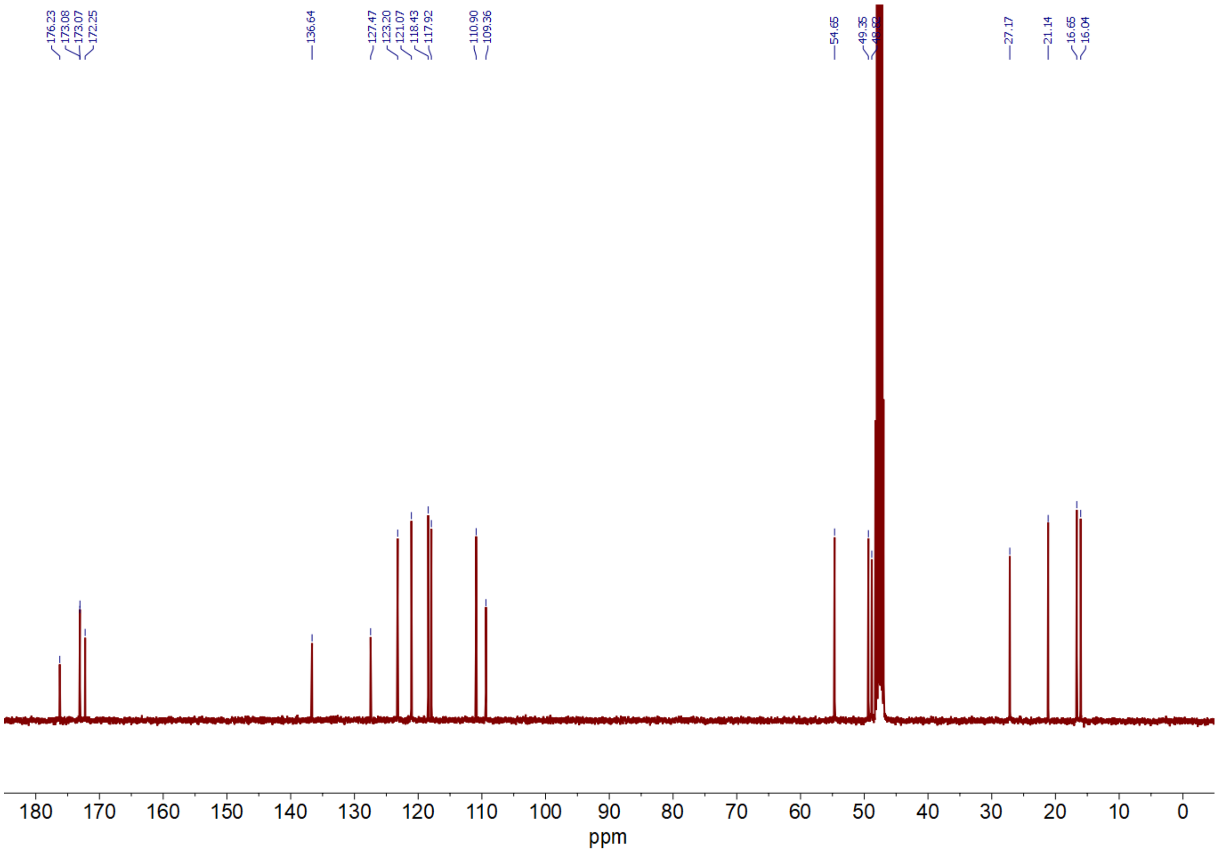

^{1}H NMR spectrum – peptide **6** (CD_3OD, 400 MHz)

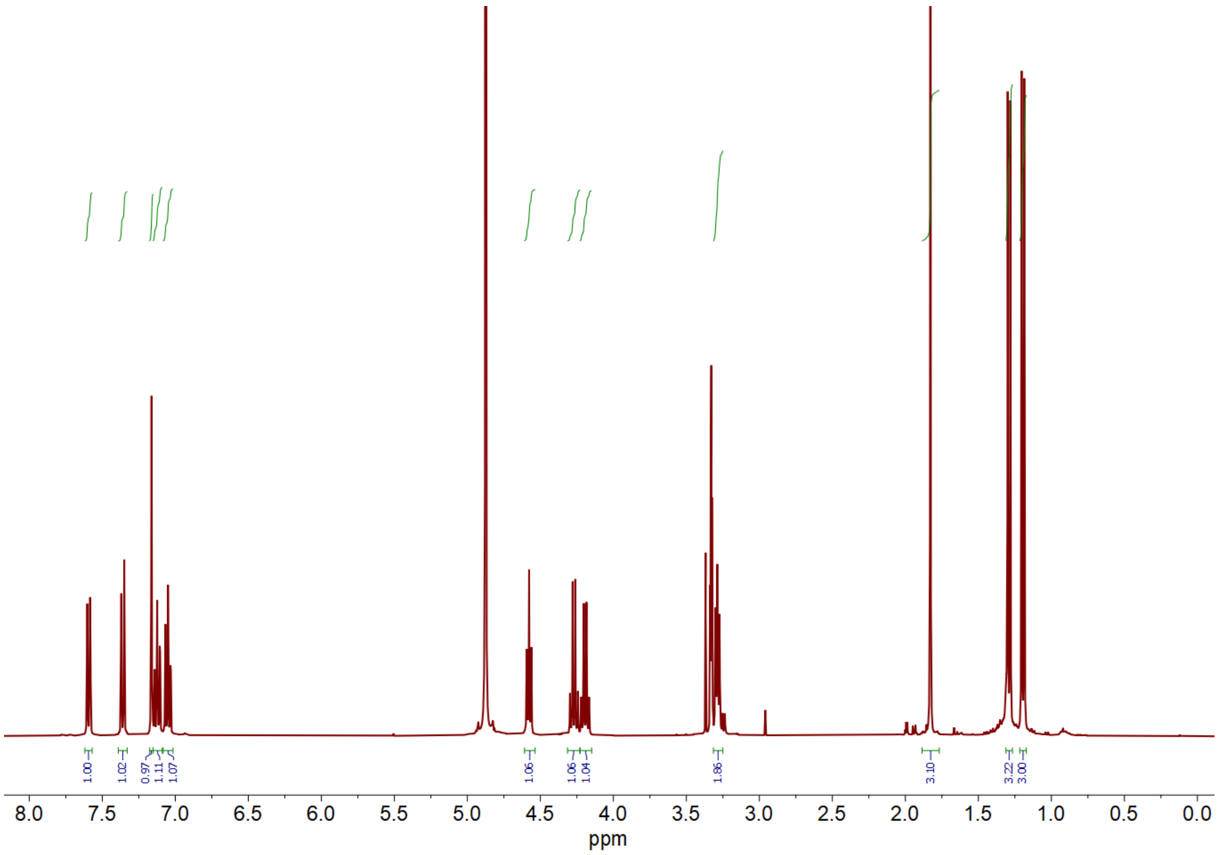

^{13}C NMR spectrum – peptide **6** (CD_3OD, 101 MHz)

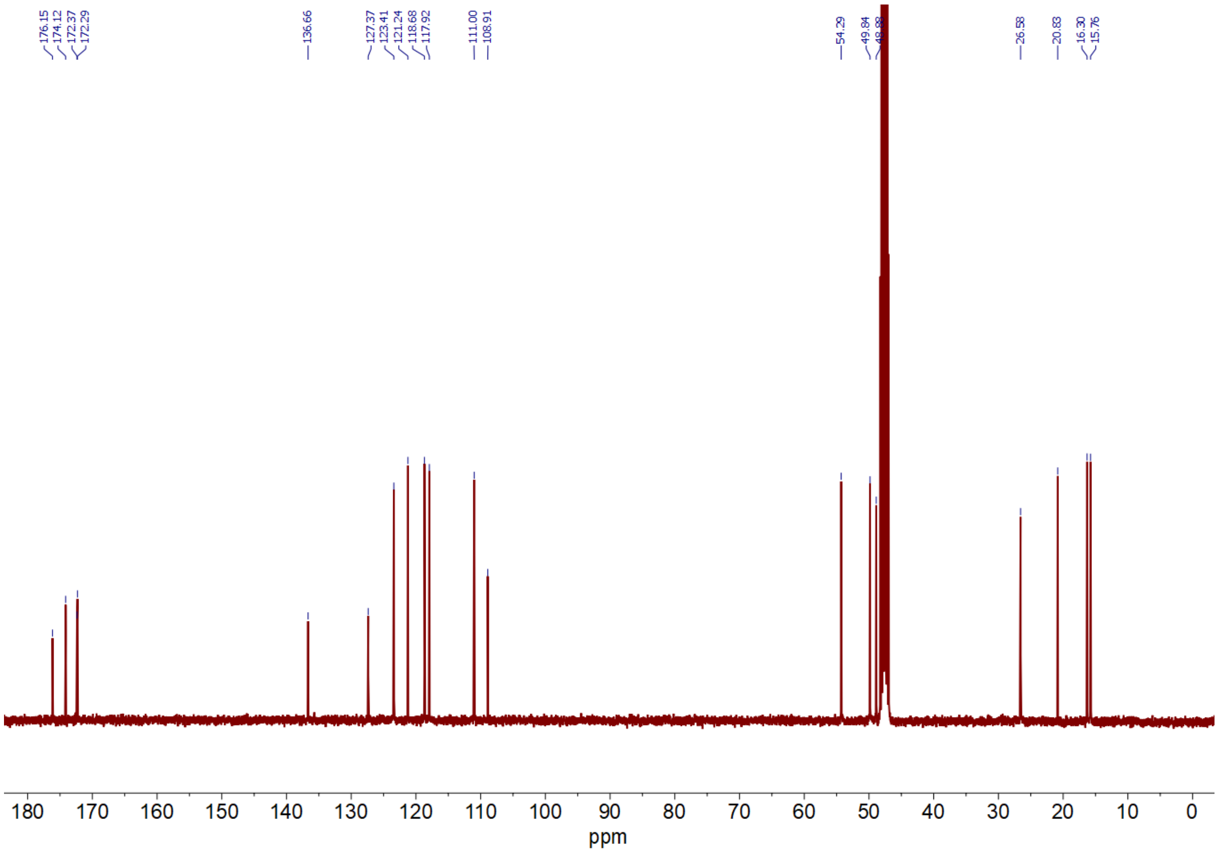

^{1}H NMR spectrum – peptide **7** (CD_3OD, 400 MHz)

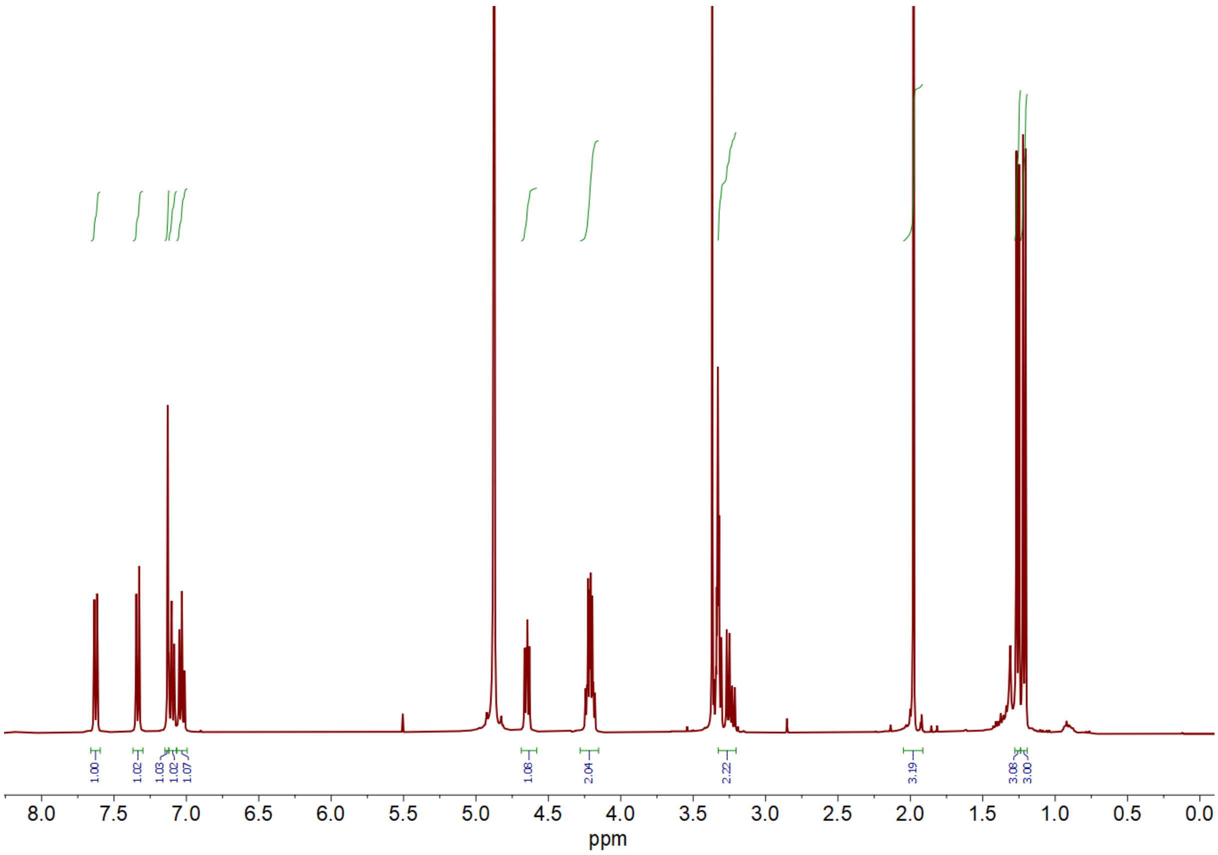

^{13}C NMR spectrum – peptide **7** (CD_3OD, 101 MHz)

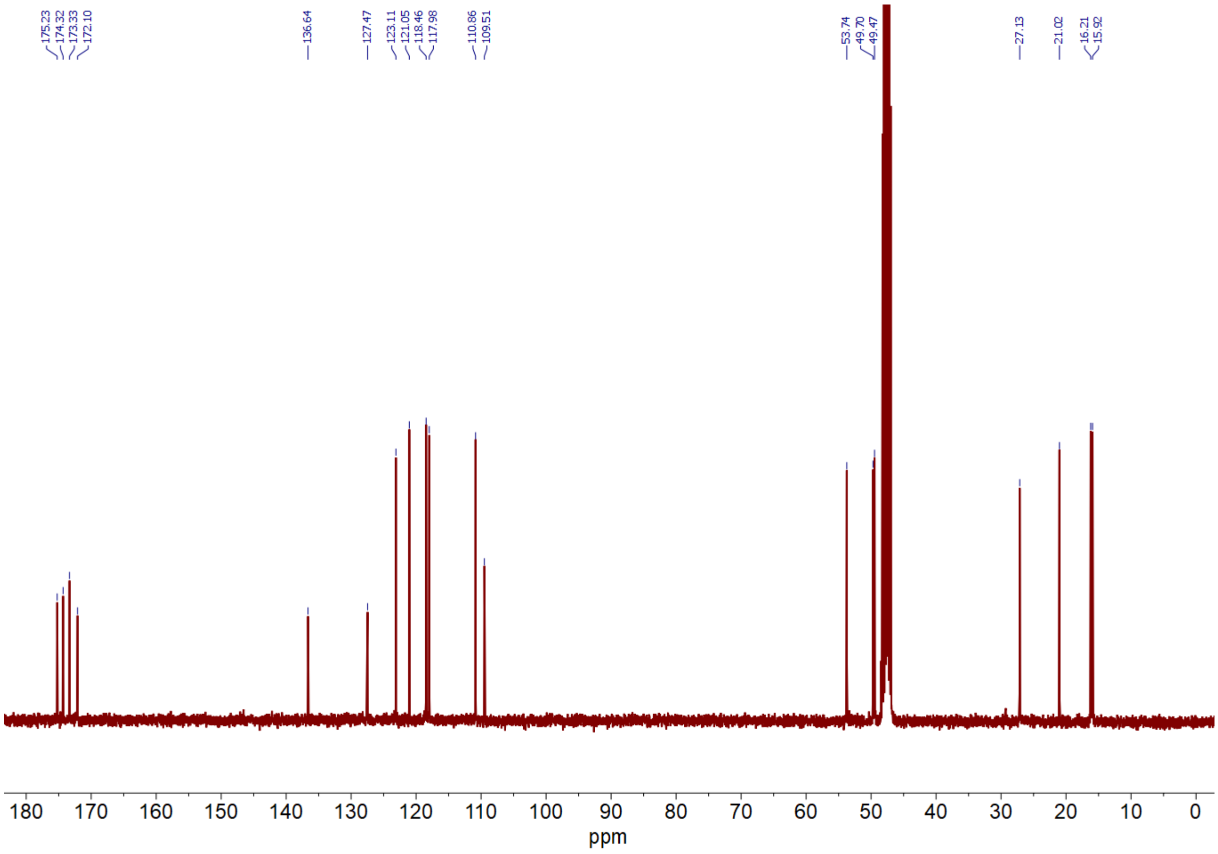

^{1}H NMR spectrum – peptide **8** (CD_3OD, 400 MHz)

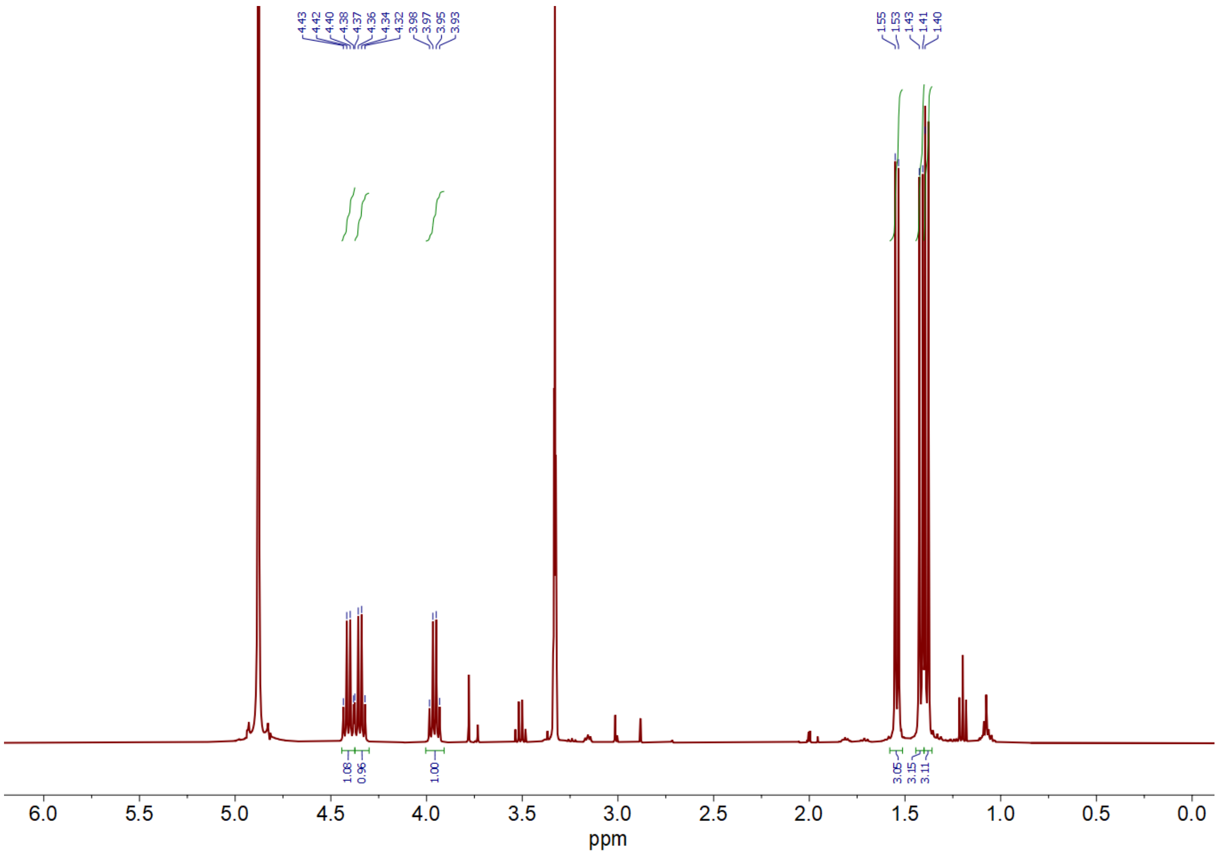

^{13}C NMR spectrum – peptide **8** (CD_3OD, 101 MHz)

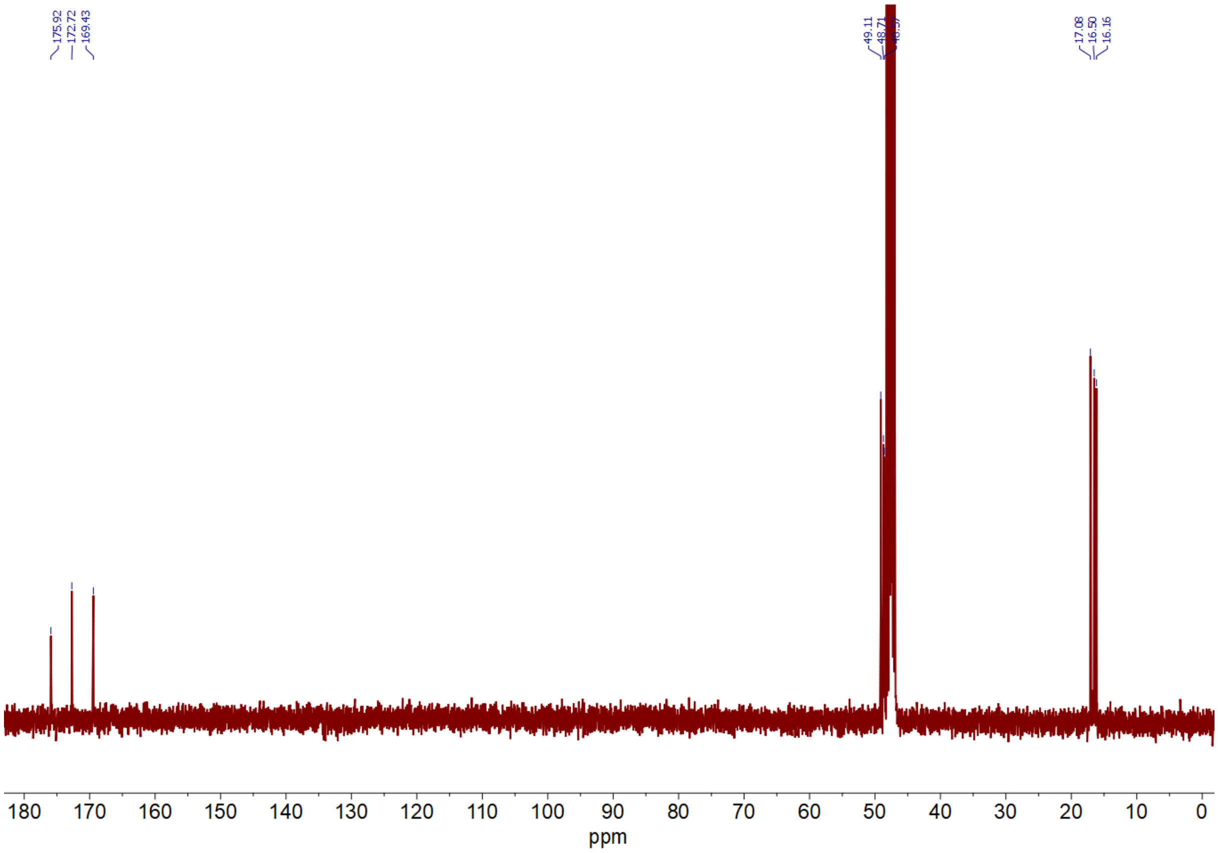

Supplement: File 1 — Materials and instruments, synthetic protocols for preparation of peptides 2–8, details of ITC, and fluorescence and NMR experiments. [file Beilstein_J_Org_Chem-21-541-s001.pdf]
